# Design and Analysis of an Upper Limb Rehabilitation Robot Based on Multimodal Control

**DOI:** 10.3390/s23218801

**Published:** 2023-10-29

**Authors:** Hang Ren, Tongyou Liu, Jinwu Wang

**Affiliations:** 1 School of Health Science and Engineering, University of Shanghai for Science and Technology, Shanghai 200000, China; renhang12138@163.com; 2 School of Biomedical Engineering, Shanghai Jiao Tong University, Shanghai 201100, China; liutongyou@sjtu.edu.cn

**Keywords:** upper limb rehabilitation robot, sEMG, kinematics analysis, joint angle

## Abstract

To address the rehabilitation needs of upper limb hemiplegic patients in various stages of recovery, streamline the workload of rehabilitation professionals, and provide data visualization, our research team designed a six-degree-of-freedom upper limb exoskeleton rehabilitation robot inspired by the human upper limb’s structure. We also developed an eight-channel synchronized signal acquisition system for capturing surface electromyography (sEMG) signals and elbow joint angle data. Utilizing Solidworks, we modeled the robot with a focus on modularity, and conducted structural and kinematic analyses. To predict the elbow joint angles, we employed a back propagation neural network (BPNN). We introduced three training modes: a PID control, bilateral control, and active control, each tailored to different phases of the rehabilitation process. Our experimental results demonstrated a strong linear regression relationship between the predicted reference values and the actual elbow joint angles, with an R-squared value of 94.41% and an average error of four degrees. Furthermore, these results validated the increased stability of our model and addressed issues related to the size and single-mode limitations of upper limb rehabilitation robots. This work lays the theoretical foundation for future model enhancements and further research in the field of rehabilitation.

## 1. Introduction

The goal of rehabilitation is to improve deficits in motor control in patients through strengthening exercises for the affected limb. Traditional rehabilitation therapy relies on one-on-one training with a rehabilitator focusing on the relearning of lost or weakened functional movements [1], which can increase staff costs and is susceptible to human factors. However, the integration of robotic technology and artificial intelligence has led to a new paradigm in rehabilitation training. In particular, upper limb rehabilitation robots, combined with industrial robots and AI, have become an important means of addressing these challenges. Patients can leverage rehabilitation robots to efficiently finish repeated exercises while collecting real-time data on themselves [2]. Since their introduction to the medical field in the 1990s, various types of structural and functional upper limb rehabilitation robots have been developed [3]. These robots can be divided into two types based on their traction contact: exoskeleton and end-effector robots.

The end-effector type rehabilitation training robot predominantly employs a series of mechanical structures or a linkage mechanism as its core framework. This system is securely affixed to the human arm through the end-effector. Furthermore, it exerts traction force on the extremities of the patient’s limbs, facilitating upper limb rehabilitation training [4,5,6,7,8].

Exoskeleton rehabilitation robots are wearable devices with mechanical structures that correspond to human joint movements. Unlike end-effector type upper limb rehabilitation robots [9], which have no fixed matching relationship with human joints, exoskeleton rehabilitation robots can effectively restrict the compensatory movements of the limb [10,11,12]. These robots achieve rehabilitation by providing traction and support to the upper limb hand, wrist, and shoulder joints. Exoskeleton solutions designed for actively guided motions usually consist of tandem links driven by powered joints. These exoskeletons and the user are connected through one or more interaction ports [13]. However, they have coaxial joints and limb joint axes, and their mechanical structure has more contact points with human joints, increasing the difficulty of human–computer interaction and control strategies and reducing the diversity of the exoskeleton mechanical structures [14,15,16,17,18]. Despite these challenges, exoskeleton rehabilitation robots are still a focus of research.

The parameters of traditional controllers are fixed and cannot be adapted to changes in the environment or tailored to the patient’s condition. In their study, Islam et al. introduced a seven-degree-of-freedom rigid upper limb exoskeleton robot that effectively stabilized the patient’s rehabilitation movements [19]. However, their control strategy remained passive, lacking the active involvement of the patients during the intermediate and late stages of rehabilitation. In a different approach, Paolucci et al. developed the MOTOR rehabilitation system [20], which allowed patients to perform translational movements with their hands in a two-dimensional horizontal plane. They incorporated trajectory planning and gamified training to enhance patient engagement and visualize the training process. Nevertheless, the system’s limited degrees of freedom restricted its applicability to horizontal plane movements, and it could not effectively address rehabilitation in the sagittal plane. Furthermore, Zhang [21] designed a novel four-degree-of-freedom upper limb rehabilitation robot, enabling training movements for the shoulder and elbow joints. They also created a virtual reality rehabilitation training system using position sensors and Unity3D(2021.3.22f1c1). However, the robot’s steel body material hindered mobility and added to the cost, making it less suitable for daily use. Additionally, the control unit’s complexity created operational inconveniences and lacked wrist joint training. In summary, none of these upper limb robots fully met the rehabilitation requirements of the patients at different stages.

Given the variations in the structure, control mechanisms, and specific treatment approaches employed in upper limb rehabilitation robots, the accurate provision of torque tailored to individual patient movements has emerged as a focal point in contemporary rehabilitation robotics research. The response of users to additional torque necessitates the precise implementation of algorithms. Achieving precise torque delivery requires the incorporation of key parameters, such as joint positions, velocities, accelerations, and kinetic modeling of the impaired limb, into the control system. Presently, upper limb exoskeleton rehabilitation robots are directed towards addressing the following challenges.

1. Enhancing patient comfort and safety: Traditional gear-driven structures tend to be bulky. The implementation of wearable rehabilitation robots employing flexible and elastic materials can reduce the weight of upper limb devices, thereby improving patient comfort and safety.
2. Diversifying rehabilitation training: Conventional systems often offer a limited range of motion, primarily focused on anterior movements, which might provide inadequate stimulation for central nervous system rehabilitation. A dynamic robot controller, adaptable to the patient’s specific rehabilitation needs and progress, along with the design of effective training modes and their corresponding hardware implementations, are critical components of robot-assisted rehabilitation.
3. Addressing joint movement uncertainty: Controlling the movement of specific muscles during the joint movement process of the affected limb can be challenging. When patients experience reduced grip strength, it’s essential to select a structural model that is better suited for the arm and hand.
4. Enhancing portability and versatility: Many exoskeleton rehabilitation robots are hindered by complex structures and limited portability, primarily due to the reliance on motor technology with inherent power constraints.

To address this, portable multifunctional rehabilitation robots should aim to combine the advantages of both traditional and portable systems, all while ensuring a proper fit with the human body’s joint axes. An example of innovation in this direction is showcased in Harvard University’s work [22], which introduced a lightweight, fully portable, textile-based, soft inflatable wearable robot. This technology, transparent when unpowered, not only offers mechanical flexibility but also enables the quantitative assessment of a user’s unrestricted movement. Such advancements may pave the way for future prosthetic applications benefiting individuals with disabilities [23]. To enhance the grip strength of patients’ hands, soft robotic gloves hold the promise of augmenting user autonomy and freedom. This is achieved through their design featuring a portable belt pouch and an open palm [24].

The upper limb rehabilitation robot in this study effectively addressed several key limitations, as follows.

1. Enhancing patient safety by positioning the motor without direct contact with the body, eliminating the need for a traditional gear-driven structure and thereby reducing the weight of the upper limb device.
2. Implementing multimodal control to better cater to the rehabilitation requirements of hemiplegic patients at various stages, ultimately reducing the workload of rehabilitators while enabling easier data acquisition and visualization.
3. Mitigating joint movement uncertainty by implementing multiple safety measures throughout the entire system, ensuring the patient’s safety from software to hardware levels.
4. Embracing a modular design for simplified disassembly and maintenance, with 3D printed components to enhance portability and provide a foundation for future personalized design enhancements.

The rehabilitation process can be categorized into three primary phases, which depend on the time elapsed since the traumatic event. These phases include the acute, subacute, and chronic phases. During each of these phases, rehabilitation therapy should facilitate the gradual recovery of the range of motion and muscle strength in the injured limb. The control strategy employed by the robotic system should be adapted to the specific needs of each phase. In the initial phase, for instance, when the patient has lost a significant portion of arm function, the robot should assist the patient in following a predetermined trajectory to enhance limb mobility and mitigate muscle atrophy or tendon retraction.

Traditional rehabilitation strategies have typically prioritized proximal limb joint rehabilitation, as nerve recovery often progresses from the proximal limb segment to the distal limb segment. However, this approach may limit opportunities for distal limb control using synapses. Bilateral coordinated movement of the upper limb is an effective neural remodeling method for stroke patients who generally suffer from unilateral hemiparesis. This method involves using the healthy limb to guide the rehabilitation training of the affected limb. When training hemiplegic patients, it is important to increase human–computer interaction as much as possible. This can enhance the motivation for training and visualize the training process.

Unlike passive robots that constantly direct the movement of the affected limb, assistive robots provide assistance only when the patient intends to initiate the movement. Both passive and assistive modes are designed for the initial stages of stroke rehabilitation, when the patient may lack the necessary strength to independently move the affected limb. On the other hand, active and resistive modes are employed in the later phases of rehabilitation as they necessitate active movement from the patient.

Facilitating a seamless human–robot interaction (HRI) that feels natural presents a significant challenge in the development of robotic rehabilitation systems. The chosen approach for detecting user intentions plays a pivotal role for ensuring a transparent and user-friendly HRI. Certain robotic rehabilitation platforms adopt bio-signals as a source for recognizing intentions [25,26,27,28,29]. Among these, electromyographic (EMG) signals are widely utilized due to their strong correlation with human motion. These signals capture the electrical activity produced by skeletal muscles responsible for executing the intended actions [30]. There is another branch of robot-assisted rehabilitation that incorporates various therapeutic approaches alongside upper extremity exoskeletons. For instance, functional electrical stimulation (FES) has demonstrated its ability in facilitating recovery in the paralyzed arms of stroke survivors. Combining functional electrical stimulation with the patient’s remaining voluntary effort has proven to enhance cortical plasticity [13].

Despite the development of numerous prototypes, research on robotic devices for upper extremity rehabilitation remains a growing field and requires new approaches to address key limitations in hardware design. In this study, we have designed an upper extremity exoskeleton robot to tackle some of the key limitations in existing exoskeletons. We have also developed a myoelectric angle synchronization acquisition system to enable more accurate myoelectric control of joint movements. The main contributions of this study are as follows.

1. The aim of this study was to conceptualize, analyze, construct, and validate a six-degree-of-freedom upper limb rehabilitation robot that seamlessly integrates EMG signal monitoring, PID control, and Unity3D simulation games. This comprehensive approach set a notable precedent for similar inquiries in the field of rehabilitation research.
2. This paper devised an eight-channel sEMG acquisition system to facilitate passive control of the affected side using an elbow rehabilitator. The feasibility of sEMG-based elbow angle prediction was validated using a back propagation neural network (BPNN).

## 2. Overall Design Solution of the Robot Mechanical Structure

### 2.1. Human Upper Limb Structure and Kinematics

In this work the interrelationships between bones, joints, muscles and other tissues of the human upper limb were analyzed. Based on this analysis and relevant clinical requirements, this paper proposed an overall design scheme for the mechanical structure of an upper limb hemiplegic rehabilitation training robot.

The human upper extremity includes the shoulder, upper arm, forearm, and hand. The main joints of the upper limbs are the sternoclavicular joint, the shoulder joint, the elbow joint, and the hand joint. The palm of the hand has a complex structure and flexible movements and is often analyzed and designed separately for rehabilitation training systems. However, the research in this paper focused on the shoulder, elbow, and wrist joints of the human upper extremity. The seven degrees of freedom of movement of the human upper limb, including the shoulder, elbow, and wrist joints, involve different muscle groups and joint mobility [31]. The relevant information for each degree of freedom is presented in Table 1. In the absence of muscular non-rigidity in the upper limb, the musculoskeletal relationships can be effectively described using the Hill model. This model predicts moments within the range of 0–30 N∙m for the shoulder and upper arm muscles, while moments within the range of 0–7 N∙m are expected for the forearm muscles [32].

### 2.2. Design Analysis of the Upper Limb Rehabilitation Robot

It has been noted that the brain plasticity theory highlights the significance of stimulating compound movements of multiple joints and muscle groups for neural recovery. Additionally, the correction of abnormal movement patterns after hemiplegia necessitates the coordination of movements among joints. As a result, single-joint or few-joint motor training alone may not be sufficient for rehabilitation. Therefore, the robot design aimed to facilitate the participation of multiple joints of the upper limb in the training simultaneously. By doing so, better rehabilitation outcomes could be achieved.

The scheme of the exoskeleton was designed by first making certain simplifications to the models of the joints.

The shoulder joint was simplified as a ball-and-socket joint with a fixed center of rotation.

The elbow joint was simplified as a hinge joint rotating around a coronal axis, providing one degree of freedom for flexion/extension.

The forearm joint was simplified as a carpel joint with one degree of freedom, which allows for inward/outward rotation around the central axis of the forearm.

Similarly, the wrist joint was simplified as an elliptical joint with two degrees of freedom: flexion/extension and retraction in the frontal and sagittal planes, respectively.

**Figure 1:**
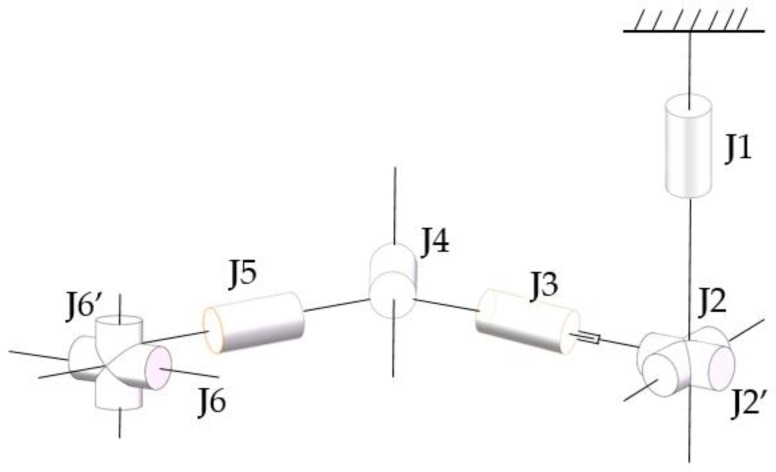
Sketch of the degrees of freedom of the exoskeleton robot.

The degrees of freedom corresponding to each joint of the human upper limb are shown in Figure 1.

Flexion/extension of the shoulder joint (J2); internal/external rotation (J1).

Flexion/extension of the elbow joint (J4).

Internal/external of the forearm (J5/J3/J2′).

Flexion/extension of the wrist joint (J6); adduction/abduction (J6′).

In addition, the sizes of the specific parts of the mechanism are required to be adjustable to accommodate different sizes of the affected limb.

## 3. The System of Rehabilitation Training

### 3.1. Development Procedure

The subsequent subsections provide comprehensive insights into the design and development particulars of the upper extremity rehabilitation robot outlined in this paper. Furthermore, Table 2 compiles the design specifications and the chosen components for the robot’s development.

During the prototype design phase, lightweight materials that were safe and reliable in terms of strength were required for the exoskeleton robot. In order to select the appropriate materials, a stress check was performed on the non-standard parts, and the maximum stress was calculated as the basis for the material selection. The torque required for shoulder joint movement was large to prevent shaking of the base during training, and the material selected for both the base and robot arm was chosen with patient safety and comfort in mind. Alloy steel was chosen for the base material, while 3D printing technology was used for the robot arm; specifically photosensitive resin and PLA were selected for their light weight and high strength. The robot arm cross-section was rounded to better fit the patient’s upper limb and increase wearing comfort.

**Figure 2:**
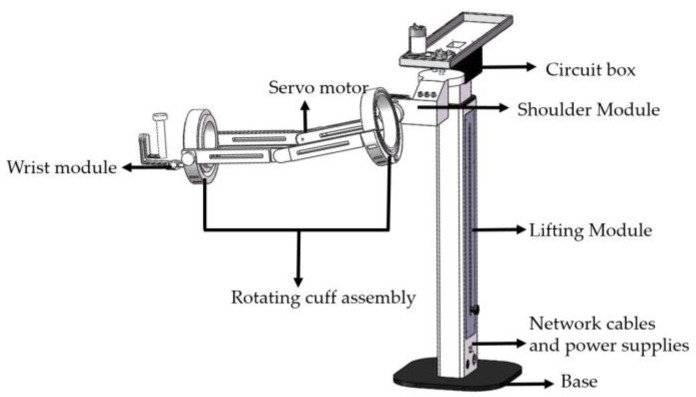
The 3D model of the upper limb rehabilitation robot.

**Figure 3:**
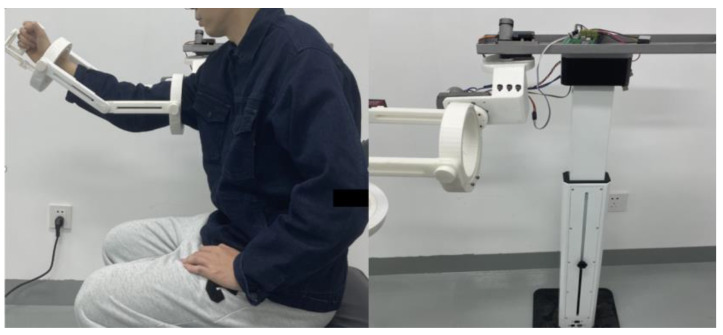
Upper rehabilitation robot (**right**) and wearing figure (**left**).

The rehabilitation robot system is depicted in Figure 2 and Figure 3.

**Figure 4:**
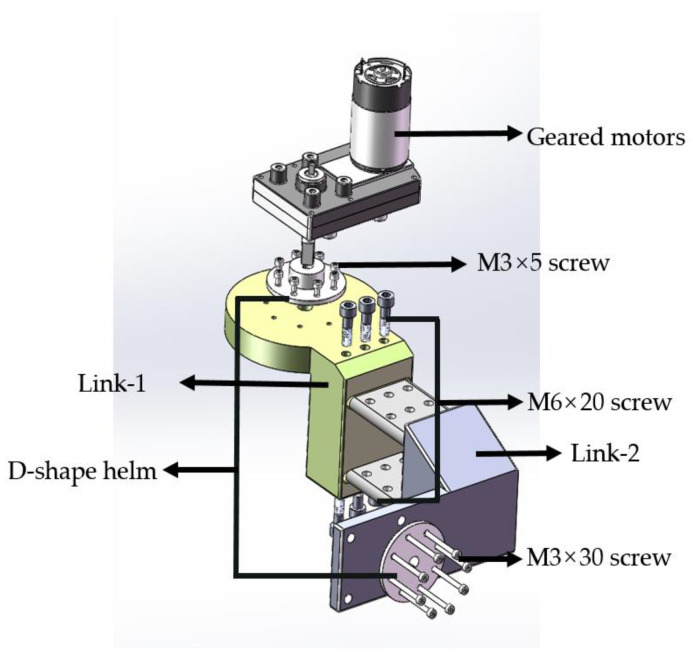
Shoulder rotation module.

For the shoulder module, to achieve both inward/outward shoulder rotation and flexion/extension, the robot’s shoulder structures were interconnected through a tandem configuration, as depicted in Figure 4. In the cross-sectional view of the frontal mechanism, the primary structures, Link-1 and Link-2, were fabricated using 3D printing, with standard components such as geared motors, a D-shaped helm, and screws. The ergonomic shoulder module was designed with two complementary structures responsible for abduction internalization (Link-2) and internal rotation externalization (Link-1). The Link-2 module features a total of 12 threaded holes—6 on the inside and 6 on the outside. These correspond to the 6 parallel holes recessed in the Link-1 module and can be securely fastened using M6 screws. Two rows of M6-threaded holes, spaced 10 cm apart, were added to accommodate patients with broader shoulders. The design of the grooves and protrusions facilitated the connection, fitting, and transmission of motion and force between the two parts. The utilization of a D-shaped rudder plate as a connector increased the contact area between the reduction gear and the rotating shaft, enhancing the stability.

**Figure 5:**
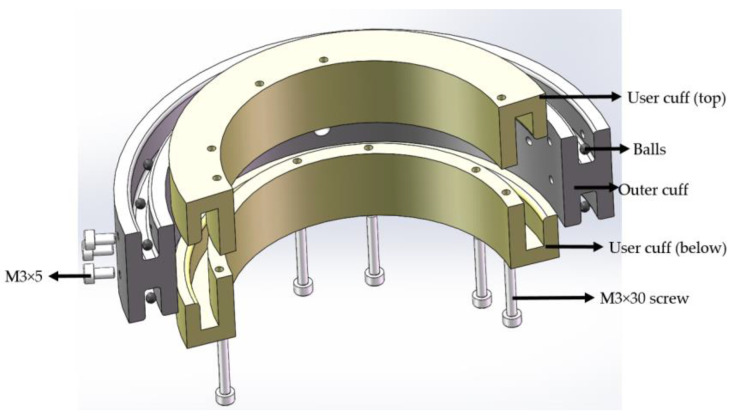
Rotating sleeve assembly.

Figure 5 displays a cross-sectional view of the rotating cuff assembly for the upper arm. To enable the large arm and forearm to rotate internally and externally, the outer cuff remained stationary while the inner cuff rotated. The outer cuff was affixed to the large and small arms of the robot’s upper limb using M3 screws. Given that the axis of rotation was situated at the humerus during forearm rotation, the ergonomic mounting position ensured a better fit around the upper limb and reduced jitter during wear and training.

In this study, the rehabilitation robot was equipped with 140 mm and 80 mm rotating sleeve assemblies on the large and small arms, respectively. Both assemblies shared the same parameters except for the varying inner and outer diameters. The inner sleeve functioned as a rotary axis, with the bearing design serving as a reference in the design. The user sleeve was divided into upper and lower sections, with a middle groove that fit into the inner convex groove of the outer sleeve band, incorporating a ball in the middle to reduce friction. M3 screws were used to join the three parts into a single unit, with a total height of 42 mm. The slide groove had a width of 5.5 mm, the convex groove was 5 mm wide, and both sides featured 1 mm rounded corners, with the ball measuring 3 mm in diameter. The entire assembly was constructed from PLA material, with a weight of 220 g for the large arm cuff assembly and 180 g for the small arm cuff. A pressure sensor was inserted between the outer cuff and the user cuff, positioned at the uppermost part of the rotating cuff, primarily intended for measuring the force of the upper limb movement along the anterior sagittal plane.

**Figure 6:**
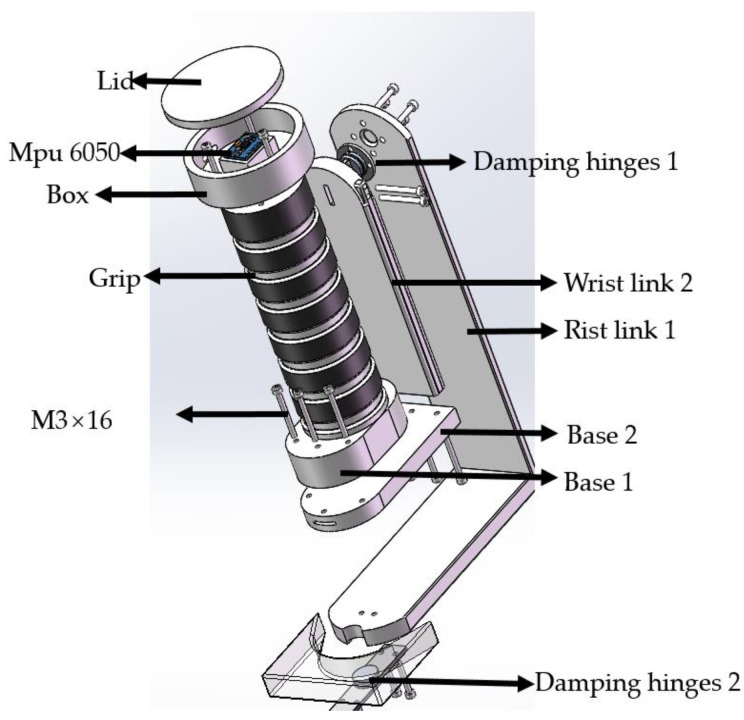
Wrist rotation assembly for the robot.

The wrist joint module of the robot consisted of two rotary joints for radial offset and flexion/extension of the wrist joint, as depicted in Figure 6. Wrist link 1 took the form of an L-shaped structure, serving as an intermediary for the connection of the wrist modules. Damping hinges 1 linked Wrist link 1 to Wrist link 2, while Damping hinges 2 connected Wrist link 1 to the primary structure of the robot. Both damping hinges shared a common solid pivot axis with a phosphor bronze pivot and offered 0.7 N·m of damping. The key distinction between the two was that Damping hinges 1 possessed a vertical structure on both sides of the pivot axis, whereas Damping hinges 2 featured a zigzag structure with symmetrical rectangular sections on either side.

In the hand module, we distinguished three primary components: the wiring box, the grip, and the base. The wiring box housed the MPU6050 posture sensor. Since there were no holes in the lid, the circuitry extended from the bottom of the box to the base of the grip. Base 1 was affixed to Base 2, and Base 2 was securely connected to Wrist link 2 using M3 screws.

In this paper, we utilized motor drives as our method of choice. To minimize any burden on the patient, the entire weight of the robot arm was attached to the shoulder module, and the base was connected to the robot arm to counteract the effect of gravity on the patient. The base’s underside featured a power on/off button, a charger port, and a network cable port. Additionally, to accommodate patients of varying heights, the base was equipped with a height-adjustable mechanism that could be modified using the knob. In order to ensure the safety of upper limb training, the motor and the corresponding upper limb mechanical joint were affixed to the movable joint through an intermediary sleeve.

In the software of the upper limb rehabilitation robot, users could select between active and passive modes and adjust the angular speed of movement, movement time, and movement angle using the interface buttons.

### 3.2. Mass and Inertia Properties of the Proposed Exoskeleton Robot

The mass, center of gravity, and inertia tensor for each component of the proposed exoskeleton robot were calculated using the Solidworks software (SolidWorks 2021). The results are presented in Table 3, categorized by the modules.

### 3.3. Safety

Safety is of paramount importance in the design of upper limb exoskeleton robots, as they operate in close proximity to the wearer. Human–robot interactions should be meticulously engineered to ensure safe operation. Achieving this safety entails the incorporation of safeguards at multiple levels, including mechanical, electronic, and control designs, to guarantee the secure usage of the robot.

On a mechanical level, safety can be established by integrating physical blocking devices within the robot’s structure. These devices serve to restrict the robot from surpassing predetermined ranges of motion (ROM). Additionally, the design of links and robot components should be such that adjacent links naturally act as physical blocking devices within the defined ROM. Furthermore, it’s crucial to regulate the robot joints by setting appropriate current and voltage limits to ensure they remain within the permissible ROM.

In terms of the electronic design, controls can be established to limit torque, force, velocity, and position through saturation mechanisms. This ensures the wearer’s safety in the event of a robot malfunction. The control algorithm employed in the rehabilitation robot discussed in this paper includes predefined thresholds for the ROM, velocity, force, and torque. These thresholds can be easily adjusted and set by users via the graphical user interface embedded in the onboard software. Additionally, in the event of an emergency or system failure, the onboard software and power button allow for a quick shutdown with a single click. By implementing these safety measures across the mechanical, electronic, and control domains, the safety of human–robot interactions is enhanced.

## 4. Kinematics, Jacobian, and Dynamics

### 4.1. Kinematic Analysis of Robots

For serial actuators, the commonly used approach by researchers is the Denavit–Hartenberg (D–H) parametric method. This method is favored due to its simplicity and ease of application in various scenarios, such as the development of forward kinematics, inverse kinematics, Jacobian, and dynamic models.

**Figure 7:**
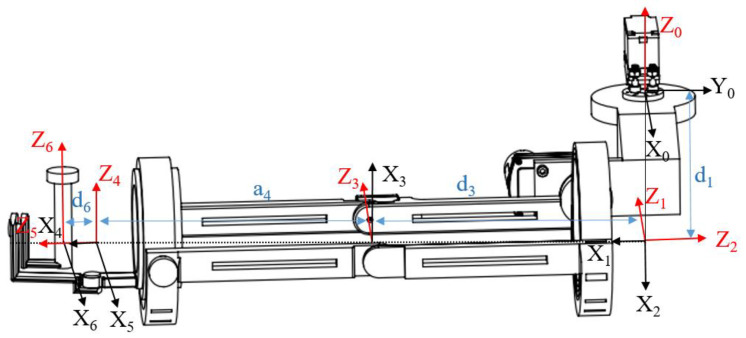
Coordinate systems of the 6-DOF robot (The red arrows denote the *Z*-axis, which is maintained perpendicular to the black arrows representing the *X*- and *Y*-axes. The blue double arrows indicate the linkage lengths or offsets of the adjacent joints).

As shown in Figure 7, the coordinate system of the robot base (base coordinate system) is represented by X_0_Y_0_Z_0_, which includes the coordinate system of joint one, followed by the coordinate systems of joints two to six. The coordinate system of the end-effector of the robot arm is represented by X_6_Y_6_Z_6_.

After establishing the joint coordinate system, the D–H parameter (Table 4) of each joint was determined using the adjacent joint coordinate system. The relationship between two adjacent links was represented by four parameters: the joint angle ***θ***, link length ***d***, link offset ***a***, and twist angle ***α***.

The general formula for the transformation between adjacent coordinates in the D–H method, which describes the transformation of coordinate system *i*−1 with respect to coordinate system *i*, is as follows.

(1) $${}_{i}^{i-1}T=A_{i}=R\left(z,\theta_{i}\right)\times T\left(0,0,d_{i}\right)\times T\left(a_{i},0,0\right)\times R\left(x,\alpha_{i}\right)$$

where *R*(*z*,*θ_i_*) and *R*(*x*,*α_i_*) are the basic rotation transformation matrices around the *z*-axis and *x*-axis, respectively, and *T*(0,0,*d_i_)* and *T*(*α_i_*,0,0) are the basic translation transformation matrices along the *z*-axis and *x*-axis, respectively. The four basic transformation matrices are all invertible matrices, so *A_i_* is also invertible. *A_i_* can be further expressed as follows.

(2) $$A_{i}=\left[\begin{array}{cccc} {\cos\;\theta }_{i} & -{\sin\;\theta }_{i}{\cos\;\alpha }_{i} & {\sin\;\theta }_{i}\;{\sin\;\alpha }_{i} & a_{i}{\cos\;\theta }_{i}\\ {\sin\;\theta }_{i} & {\cos\;\theta }_{i}{\cos\;\alpha }_{i} & -{\cos\;\theta }_{i}{\sin\;\alpha }_{i} & a_{i}{\sin\;\theta }_{i}\\ 0 & {\sin\;\alpha }_{i} & {\cos\;\alpha }_{i} & d_{i}\\ 0 & 0 & 0 & 1 \end{array}\right]$$

The D–H parameters can be substituted into Equation (2), as follows.

$${}_{1}^{0}T=\left[\begin{array}{cccc} c_{1} & 0 & s_{1} & 0\\ s_{1} & 0 & -c_{1} & 0\\ 0 & 1 & 0 & d_{1}\\ 0 & 0 & 0 & 1 \end{array}\right],\;{}_{2}^{1}T=\left[\begin{array}{cccc} c_{2} & 0 & s_{2} & 0\\ s_{2} & 0 & -c_{2} & 0\\ 0 & 1 & 0 & 0\\ 0 & 0 & 0 & 1 \end{array}\right],\;\;{}_{3}^{2}T=\left[\begin{array}{cccc} c_{3} & 0 & s_{3} & 0\\ s_{3} & 0 & -c_{3} & 0\\ 0 & 1 & 0 & d_{3}\\ 0 & 0 & 0 & 1 \end{array}\right],\;{}_{4}^{3}T=\left[\begin{array}{cccc} c_{4} & 0 & -s_{4} & a_{4}c_{4}\\ s_{4} & 0 & c_{4} & a_{4}s_{4}\\ 0 & -1 & 0 & 0\\ 0 & 0 & 0 & 1 \end{array}\right],\;\;{}_{5}^{4}T=\left[\begin{array}{cccc} c_{5} & 0 & s_{5} & 0\\ s_{5} & 0 & -c_{5} & 0\\ 0 & 1 & 0 & 0\\ 0 & 0 & 0 & 1 \end{array}\right],\;{}_{6}^{5}T=\left[\begin{array}{cccc} c_{6} & 0 & s_{6} & 0\\ s_{6} & 0 & -c_{6} & 0\\ 0 & 1 & 0 & d_{6}\\ 0 & 0 & 0 & 1 \end{array}\right]$$

The matrix representation of the robot’s end position in the base coordinate system (kinematic equations of the robot) is given by the following.

(3) $${}_{6}^{0}T{=}_{1}^{0}T_{2}^{1}T_{3}^{2}T_{4}^{3}T_{5}^{4}T_{6}^{5}T=\left[\begin{array}{cccc} n_{x} & o_{x} & a_{x} & p_{x}\\ n_{y} & o_{y} & a_{y} & p_{y}\\ n_{z} & o_{z} & a_{z} & p_{z}\\ 0 & 0 & 0 & 1 \end{array}\right]$$

The end position matrix of the robot is as follows.

(4) $$P^{T}={\left[P_{x},P_{y},P_{z}\right]}^{T}$$

In Equation (3),

${\left[\begin{array}{ccc} n_{x} & n_{y} & n_{z} \end{array}\right]}^{T}$ is the direction vector of the X_6_-axis of the robot’s end (handle) coordinate system in the base coordinate system.

${\left[\begin{array}{ccc} o_{x} & o_{y} & o_{z} \end{array}\right]}^{T}$ is the direction vector of the Y_6_-axis of the robot’s end (handle) coordinate system in the base coordinate system.

${\left[\begin{array}{ccc} a_{x} & a_{y} & a_{z} \end{array}\right]}^{T}$ is the direction vector of the Z_6_-axis of the robot’s end (handle) coordinate system in the base coordinate system.

n_x_ = (c_4_c_5_c_6_ − s_4_s_6_)(s_1_s_3_ + c_1_c_2_c_3_) + s_5_c_6_(s_1_c_3_ − c_1_c_2_s_3_) + c_1_s_2_(s_4_c_5_c_6_ + c_4_s_6_)

n_y_ = (s_4_s_6_ − c_4_c_5_c_6_)(c_1_s_3_ − s_1_c_2_c_3_) + s_1_s_2_(c_4_s_6_ − s_4_c_5_c_6_) − s_5_c_6_(c_1_c_3_ + s_1_c_2_s_3_)

n_z_ = −c_6_c_5_(c_2_s_4_ − s_2_c_3_c_4_) − c_6_s_2_s_3_s_5_ − s_6_(c_2_c_4_ + s_2_c_3_s_4_)

o_x_ = c_4_s_5_ (s_1_s_3_ + c_1_c_2_c_3_) + s_5_c_1_s_2_s_4_ − c_5_(s_1_c_3_ − c_1_c_2_s_3_)

o_y_ = c_5_(c_1_c_3_ + s_1_c_2_s_3_) − s_5_c_4_(c_1_s_3_ − s_1_c_2_c_3_) + s_1_s_2_s_4_s_5_

o_z_ = s_2_s_3_c_5_ − s_5_(c_2_s_4_ − s_2_c_3_c_4_)

a_x_ = (s_4_c_6_ + c_4_c_5_s_6_)(s_1_s_3_ + c_1_c_2_c_3_) + s_5_s_6_(s_1_c_3_ − c_1_c_2_s_3_) + c_1_s_2_(s_4_c_5_s_6_ − c_4_c_6_)

a_y_ = (−s_4_c_6_ − c_4_c_5_s_6_)(c_1_s_3_ − s_1_c_2_c_3_) − s_5_s_6_(c_1_c_3_ + s_1_c_2_s_3_) + s_1_s_2_(s_4_c_5_s_6_ − c_4_c_6_)

a_z_ = c_6_(c_2_c_4_ + s_2_c_3_s_4_) − c_5_s_6_(c_2_s_4_ − s_2_c_3_c_4_) − s_2_s_3_s_5_s_6_

p_x_ = (a_4_c_4_ + c_4_s_5_d_6_)(s_1_s_3_ + c_1_c_2_c_3_) + c_1_s_2_d_3_ − c_5_d_6_(s_1_c_3_ − c_1_c_2_s_3_) + c_1_s_2_s_4_s_5_d_6_ + c_1_s_2_s_4_a_4_

p_y_ = (−a_4_c_4_ − c_4_d_6_s_5_)(c_1_s_3_ − s_1_c_2_c_3_) + c_5_d_6_(c_1_c_3_ + s_1_c_2_s_3_) + s_1_s_2_(s_4_s_5_d_6_ + d_3_ + a_4_s_4_)

p_z_ = d_1_ − c_2_d_3_ − s_5_d_6_(c_2_s_4_ − s_2_c_3_c_4_) + s_2_s_3_c_5_d_6_ − c_2_a_4_s_4_ + s_2_c_3_a_4_c_4_

where s_i_ stands for sin *θ_i_,* c_i_ stands for cos *θ_i_*, d_1_ = 80 mm, d_3_ = 330 mm, d_6_ = 4~20 mm, and a_4_ = 400 mm.

### 4.2. Jacobian

In this paper, the linear velocity vector of the upper limb rehabilitation robot consisted of velocities along the three Cartesian axes, and the rotational velocity vector included the angular velocities around the Cartesian axes. In Equation (5), $q_{1}$…$q_{n}$ for the joint variables, $x_{p}$ represented an output component of the multivariate function. ε was either 0 or 1; 0 when the joint was a rotating joint and 1 when it was a moving joint, and $\overset{-}{\varepsilon }$ was the inverse of ε. ${}_{}^{0}Z_{1}$…${}_{}^{0}Z_{n}$ represented the output vector of the multivariate function. The Jacobian matrix of the robot, which was a 6 × 6 matrix, was calculated using MATLAB (version R2018a, MathWorks, Natick, MA, USA) and based on Equation (5).

(5) $$J=\left(\begin{array}{cccc} \frac{\partial x_{p}}{\partial q_{1}} & \frac{\partial x_{p}}{\partial q_{2}} & \cdots & \frac{\partial x_{p}}{\partial q_{n}}\\ {\overset{-}{\varepsilon }}_{1}{}_{}^{0}Z_{1} & {\overset{-}{\varepsilon }}_{2}{}_{}^{0}Z_{2} & \cdots & {\overset{-}{\varepsilon }}_{n}{}_{}^{0}Z_{n} \end{array}\right)$$

### 4.3. Dynamics

The dynamic equations of the robot were derived from the Newton–Euler iterative formulation as follows.

(6) $$\tau =M(q)\vec{q}+V(q,\dot{q})\dot{q}+G(q)+F(q,\dot{q})$$

where M(q) is the 6 × 6 mass matrix of the manipulator, $V(q,\dot{q})$ is a 6 × 1 dimension vector ‘composed of the centrifugal and Coriolis terms, and G(q) is a 6 × 1 vector of the gravity terms. In addition, $F(q,\dot{q})$ is a 6 × 1 vector of nonlinear Coulomb friction and can be expressed using the following relation with a coefficient of friction c. $\mathrm{sgn}(\dot{q})$ is a signed function of $\dot{q}$’s (generalized velocity or velocity vector), indicating the direction of the velocity. The *M*, *V*, *G*, and *F* matrices were computationally intensive and, therefore, are not explained in the article.

(7) $$F(q,\dot{q})=c.\mathrm{sgn}(\dot{q})$$

## 5. Elbow Joint Angle Prediction System

sEMG not only reflects the anatomical structure and physiological properties of the muscles being monitored, but also contains information about their movement, such as the muscle moment, movement speed, and movement angle. Since sEMG is non-invasive, rapid, and convenient, it is commonly used in rehabilitation medicine to evaluate muscle function recovery. The field of prosthetic control is also based on sEMG, which is used for motion recognition and classification to control the movement of prostheses according to the patient’s intention [33,34,35].

**Figure 8:**
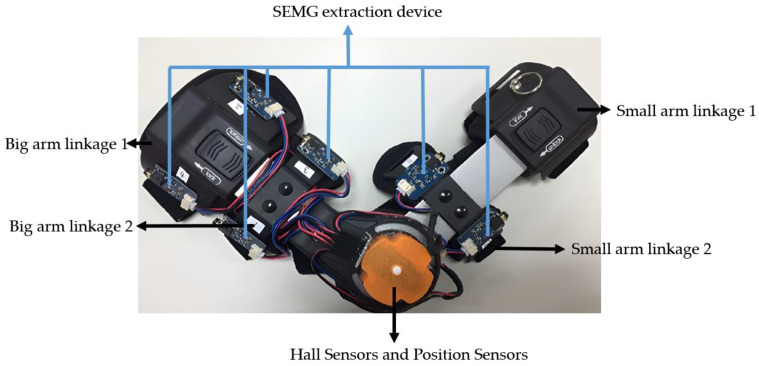
Synchronized signal acquisition equipment.

**Figure 9:**
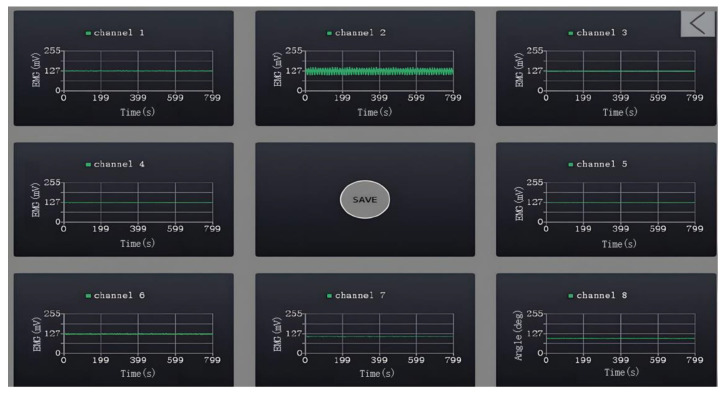
Synchronized signal acquisition system upper computer software.

In order to establish the relationship between sEMG and the elbow joint angle, it was necessary to collect joint angle data during the flexion and extension movements of the elbow joint. This information served two purposes: first, as a target value for model training and ongoing evaluation; and second, as a metric to validate the model performance and prediction accuracy. The synchronized signal acquisition system (Figure 8 and Figure 9) consisted of two parts: hardware and software. In the hardware part, the sEMG sensors were distributed around the elbow muscles and the angle sensors were located on the rotation axis.

The small arm and large arm of the rehabilitation device were encompassed by two modules, each equipped with six sEMG sensors. Data acquisition was facilitated through the use of an existing data acquisition card, which performed the essential tasks of EMG signal capture and digital-to-analog conversion at a sampling frequency of 650 Hz.

To operate the device, the white switch located at the junction of the large arm and the small arm was pressed. Upon doing so, a green light in a slow-flashing state became visible. Next, the device was brought into proximity with the matching dongle to initiate the Bluetooth connection. Once the connection was successfully established, the indicator light transitioned from a slow flash to a steady, normal light, signifying that the Bluetooth pairing process was complete.

The upper computer interface, as depicted in Figure 9, served as the central hub for the real-time display, acquisition, and storage of sEMG and the joint angle data. This interface comprised eight display channels and was equipped with an operational button situated at its center. Of the eight channels, the lower right corner featured channel 8, which was dedicated to joint angle information, presenting data regarding the bending angle of the arm. The remaining seven channels served as myoelectric signal channels, presenting real-time waveforms of the sEMG.

The central operational button held the key functions for initiating data acquisition and saving for both sEMG and the joint angle data. Prior to commencing the data acquisition, this button was displayed as the “save” button. By clicking the “save” button, the data acquisition began instantaneously, and the button transformed into a “stop” button. When the required data amount was collected, the “stop” button was clicked to halt the data acquisition, and the generated data file was saved in bin format on the computer.

### 5.1. Analytical Study of Signal Preprocessing

In order to establish a quantitative relationship between sEMG and the elbow joint angle, it was essential to collect joint angle data during elbow flexion and extension movements. This data served two purposes: one was to be used as the target value for model training and continuous optimization, and the other was to be used as an index for evaluating the model’s performance and verifying its prediction effect.

Common joint angle measurement methods fall into two categories: direct and indirect. The advantage of the direct measurement method is its low cost and simplicity of operation. However, the device needs to be bound to the limb in direct contact, which may limit limb movement. For this study, the direct measurement method was adopted using an angle sensor (Shanghai Aoyi Information Technology Co., Ltd., Shanghai, China). This sensor contained a circular rotating varistor that changed resistance value when the angle rotated, allowing for the rotation angle to be obtained by measuring the dividing voltage of one of the segments of the varistor.

EMG signals are divided into two types based on the type of electrodes used for collection: nEMG and sEMG. For this study, the surface electrode acquisition method (sEMG) was used, where surface electrodes were attached to the skin surface to pick up the superposition of the action potentials from different motor units in time and space. This method has the advantage of being non-invasive and simple to operate, and the measured EMG changes can reflect the functional changes of the whole muscle, making it suitable for sports training applications [36].

For this experiment, the selected electrode patch was a single-use ECG electrode. The EMG sensor used in this paper integrated the filtering and amplification circuits. It amplified weak human sEMG signals within the range of ±1.5 mV by 1000 times and effectively suppressed noise, especially industrial frequency interference, through differential input and analog filtering circuits. The output signal was in analog form, with 1.5 V as the reference voltage, and the output range was 0~3.0 V. The signal amplification was consistent and unchanged for all the subjects in the experiments herein.

### 5.2. Preprocessing of the EMG Signals

The acquired sEMG and joint angle signals were susceptible to noise and required preprocessing. sEMG, like other physiological electrical signals, has a low amplitude and demands precise experimental equipment and controlled environmental conditions. In this chapter, we employed the Fourier transform for sEMG noise reduction and wavelet transform for the joint angle signal noise reduction. We also analyzed the process and outcomes of signal preprocessing.

The Fourier transform noise reduction process began with transforming the time domain signal using the Fourier analysis. The transformed signal was then filtered to eliminate noise frequencies. Finally, the filtered signal underwent Fourier inversion to yield a noise-reduced signal. In the case of sEMG acquisition, the noise primarily arose from industrial frequency interference related to the equipment and inherent cardiac interference from the human body. A breakdown of the primary sources and their associated frequency ranges of noise can be found in Table 5.

As shown in the table, the noises mixed into the sEMG were primarily low-frequency noises with frequencies below 30 Hz, with the exception of the 50 Hz industrial frequency interference. The sampling frequency of the synchronous signal acquisition system was 650 Hz, which meant the acquired signal frequency range was 0–325 Hz.

To filter out noise while retaining the useful information of the original signal, we designed both a 30–300 Hz bandpass filter and a 50 Hz IF trap using the MATLAB(MATLAB R2018b) toolbox Filter Design and Analysis Tool with the ‘filterDesigner’ command. The default setting for the filter was an infinite impulse response (IIR) filter.

This paper selected the Butterworth filter for both the 30–300 Hz bandpass filter and the 50 Hz IIR trap. For the 30–300 Hz bandpass filter, we set the stopband cutoff frequencies to 10 Hz and 320 Hz, the passband cutoff frequencies to 30 Hz and 300 Hz, the stopband attenuation to 40 dB, and the passband attenuation to 1 dB. This resulted in a 10th order Butterworth bandpass filter.

For the 50 Hz trap, we set the passband cutoff frequencies to 47 Hz and 53 Hz, the stopband cutoff frequencies to 49.5 Hz and 50.5 Hz, the two passband attenuations to 5 dB and 1 dB, respectively, and the stopband attenuation to 30 dB. This yielded a sixth order industrial frequency trap.

**Figure 10:**
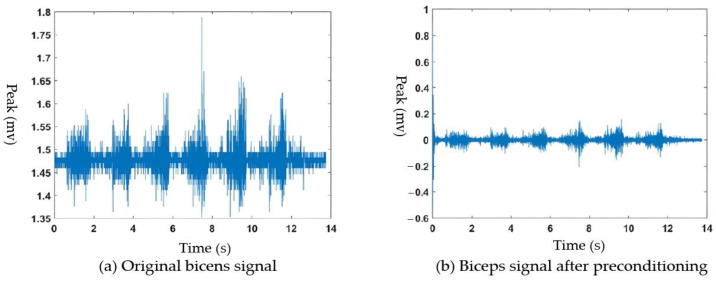
Comparison of the EMG signal waveforms before and after filtering.

Using the parameters described above, the signal was first bandpass filtered and then 50 Hz trap filtered. Figure 10a displays the waveforms of the sEMG signal from the biceps brachii muscle before and after filtering, while Figure 10b exhibits the waveform of the biceps sEMG signal after filtering.

**Figure 11:**
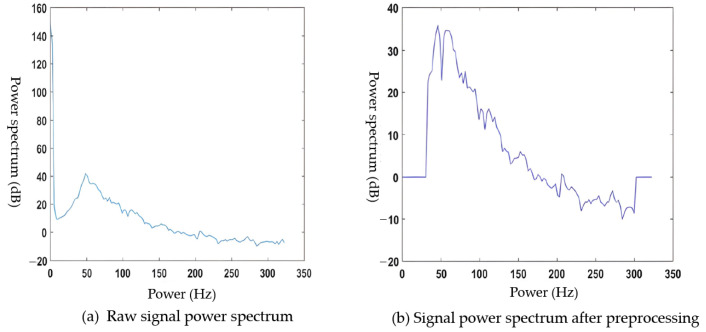
Comparison of the signal power spectrum before and after filtering.

As seen in Figure 10, the filter removed the bias voltage and the noise-induced spikes in the original sEMG, resulting in a smoother and cleaner signal waveform with the baseline amplitude fluctuating above and below 0. As shown in Figure 11, after filtering, the power spectrum outside the 30–300 Hz band registered at 0, and the power at 50 Hz frequency was significantly reduced. This demonstrated that the designed filter effectively suppressed the influence of various frequency noises on the sEMG.

For preprocessing the joint angle signals, we employed wavelet transform denoising in MATLAB. While MATLAB offers a range of wavelet denoising functions, our paper utilized the one-dimensional automatic noise reduction function, ‘wden’. To determine the threshold value and choose the appropriate threshold function, we made the following settings. We opted for the heuristic threshold calculation method to compute the threshold value and selected the soft threshold function for filtering the wavelet system.

**Figure 12:**
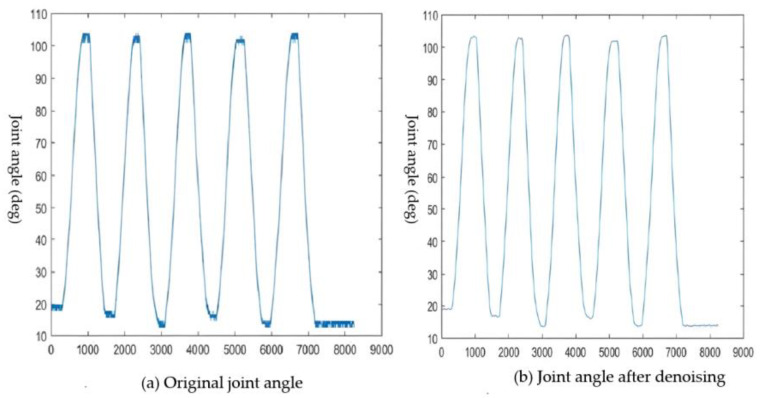
Comparison of the joint angles before and after denoising.

In order to achieve optimal denoising results, we assessed the denoising effect using the ‘smoothness r’ index. This index, set at a decomposition level of four layers, was based on the Coiflet-4 (coif4) wavelet basis. The results of this denoising process are presented in Figure 12.

### 5.3. Experimental Data Acquisition

First, the acquired sEMG and joint angle signals underwent preprocessing to cancel out any noise. Since most of the noise frequencies in the sEMG were below 30 Hz, the Fourier transform was utilized for denoising, using a bandpass filter with a range of 30–300 Hz and an industrial frequency trap of 50 Hz to effectively filter out the noise in the sEMG.

For the joint angle signal, wavelet threshold denoising was employed. The number of wavelet decomposition layers was set to four, and the coif4 function was used as the wavelet basis. This approach successfully eliminated noise in the joint angle signal using the one-dimensional automatic noise cancellation function “wden”.

Furthermore, taking advantage of the synchronous acquisition of the sEMG and joint angle signals, the starting and ending points of the muscle activity segments were detected based on the angle signal waveforms. This step laid the foundation for the subsequent extraction of the characteristics of the muscle activity segments.

The enrolled participants in the experiment were required to be in good health, without any neuromuscular diseases, and possess flexible and obstacle-free movement in all their joints. To ensure the validity of the experiment, the participants were not allowed to engage in strenuous exercise within 24 h before the data collection began, ensuring that their upper limbs were in a normal condition during the experiment. There were six enrolled participants in this study, comprising four males (coded as M1~M4) and two females (coded as W1~W2). The basic information about the enrolled participants is presented in Table 6.

In this paper, the upper limb muscles associated with elbow flexion/extension that were measured included the triceps brachii, biceps brachii, elbow, and brachioradialis muscles. To collect the EMG signals and angle signals during elbow flexion and extension, three surface electrode patches were affixed to each muscle: positive, negative, and reference electrodes.

**Figure 13:**
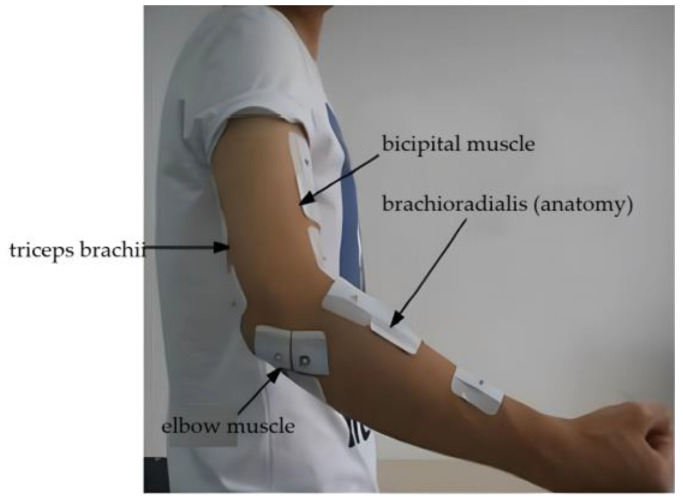
Surface electrode sticking position.

Before placing the electrodes, it was important to enhance the skin’s adherence to the electrodes by preparing the measurement area. This involved cleaning the area with mild soap and water to eliminate dirt, sweat, sebum, and other impurities. If the measurement area was covered by thick hair, it was advisable to trim or shave the hair. Long hairs could obstruct the electrodes from making close contact with the skin, potentially affecting the signal quality. Since the surface electrode patch primarily comprised a non-woven backing, pressure-sensitive adhesive, electrode core, and conductive adhesive, no additional skin preparations were required. Once the measurement area was completely dry, the electrodes were attached to the skin, as illustrated in Figure 13.

**Figure 14:**
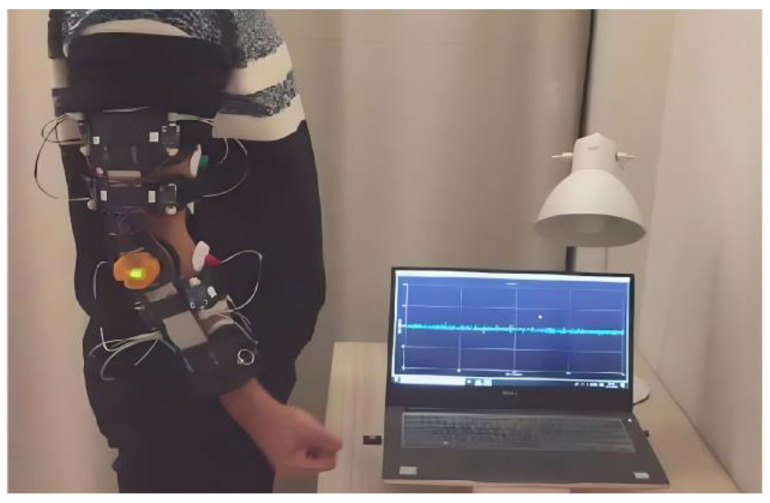
Experimenter’s wear chart. The subject stood in an upright position, with arms naturally hanging down, the forearm and upper arm forming a straight line perpendicular to the horizontal plane, and the palm of the hand clenched in a fist with the palm facing upward.

The preparatory posture at the beginning of each experiment was set as follows. The subject stood in an upright position, with arms naturally hanging down, the forearm and upper arm forming a straight line perpendicular to the horizontal plane, and the palm of the hand clenched in a fist with the palm facing upward. The synchronized signal acquisition system was worn on the subject’s right arm, as shown in Figure 14. Prior to each experiment, angular calibration was performed. The calibration method involved having the subject relax and naturally hold the preparatory posture for 10 s before the start of the experiment. The angle signal during this preparatory posture was saved and subsequently denoised and averaged to obtain the baseline value of the angle signal.

The angle data collected during the elbow flexion/extension movement was referred to as the “original angle”. To obtain the actual angle during the elbow flexion/extension movement, the baseline angle was subtracted from the original angle data. This process enabled the determination of the real angle during the elbow flexion/extension movement for further analysis.

### 5.4. Feature Extraction and Quantitative Modeling

The purpose of this chapter was to extract suitable EMG signal eigenvalues to lay the foundation for the subsequent modeling. There were two evaluation indices for the prediction effect of the model. One was the mean square error (MSE) between the predicted value and the real value. The smaller the MSE value, the better the prediction effect, as shown in Equation (8).

Another indicator is the regression coefficient (R), as shown in Equation (9). R represents the correlation between the predicted and actual values. An R value of 1 indicates a strong relationship, while an R value of 0 indicates a random relationship [36].

In summary, a smaller MSE value and a larger R value indicated a better prediction effect, as described in Equation (9). Where ${\dot{\theta }}_{i}$ is the predicted angle value of the model, $\theta_{i}$ is the actual measured angle value, and n is the number of sampling points of the sample. $\overset{-}{\dot{\theta }}$ and $\overset{-}{\theta }$ are the average of the predicted angle and the average of the actual measured angle, respectively.

(8) $$MSE=\frac{\sum_{i=1}^{n}{\left({\dot{\theta }}_{i}-\theta_{i}\right)}^{2}}{n}$$

(9) $$R=\frac{\frac{1}{n}\sum_{i=1}^{n}\left(\theta_{i}-\overset{-}{\theta }\right)\left({\dot{\theta }}_{i}-{\overset{-}{\dot{\theta }}}_{i}\right)}{\sqrt{\frac{1}{n}\sum_{i=1}^{n}{\left({\dot{\theta }}_{i}-{\dot{\theta }}_{i}\right)}^{2}}\sqrt{\frac{1}{n}\sum_{i=1}^{n}{\left(\theta_{i}-\overset{-}{\theta }\right)}^{2}}}$$

By comparing the root mean square (RMS), mean absolute value (MAV), integral absolute value (IAV), wave length (WL), Wilson amplitude (WAMP), zero crossing (ZC), slope sign change (SSC), mean power frequency (MPF), peak frequency (PKF), median frequency (MF), total power (TTP), spectral moments (SM), auto regressive (AR), and SampEn eigenvalues of the model prediction effect, we finally selected the fifth order AR model coefficients as the input values of the BP neural network. The prediction merits of the elbow joint angle were then analyzed using MATLAB with error back propagation neural network (BPNN), generalized regression neural network (GRNN), support vector machine regression (SVR), regression tree (RT), and ensemble regression learning (ERL) regression algorithms.

**Figure 15:**
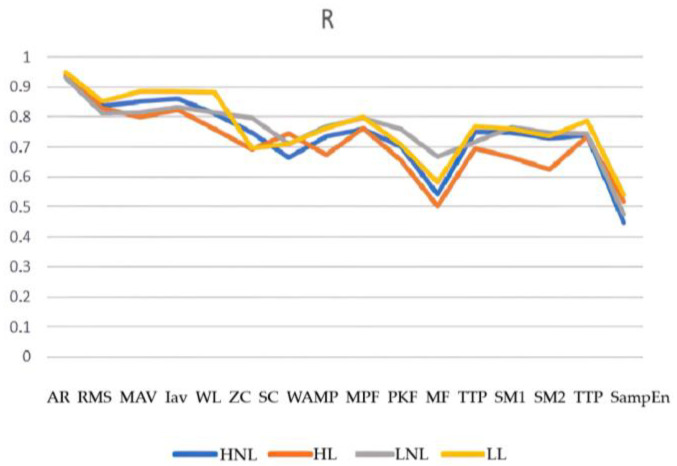
Linear regression R values for the different eigenvalues.

**Figure 16:**
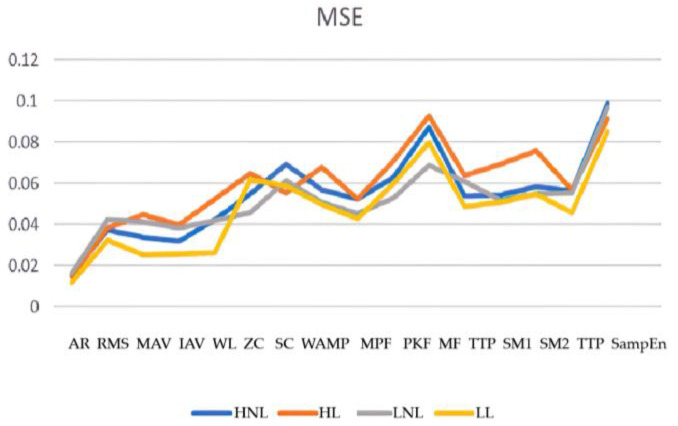
Mean square error for the different eigenvalues.

**Figure 17:**
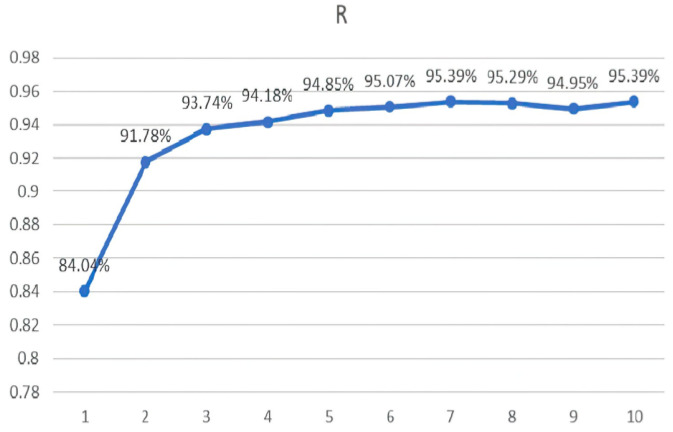
Linear regression R values for the different AR model orders.

**Figure 18:**
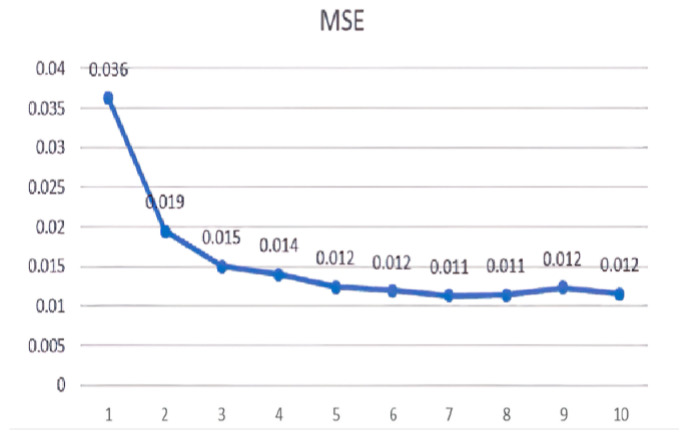
Mean square error for the different AR model orders.

**Figure 19:**
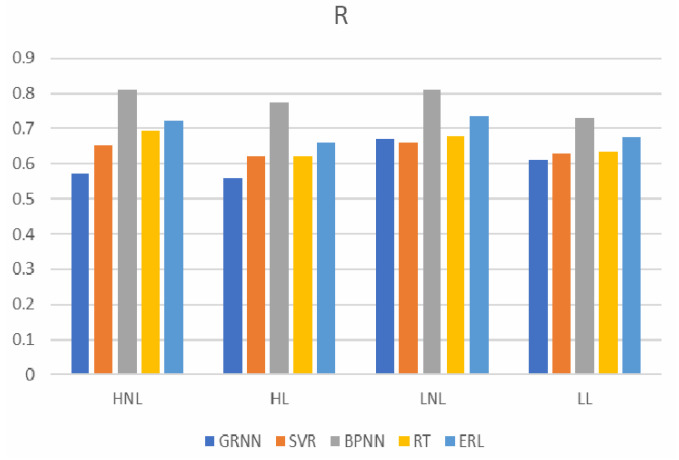
Linear regression R values for the different regression algorithms.

**Figure 20:**
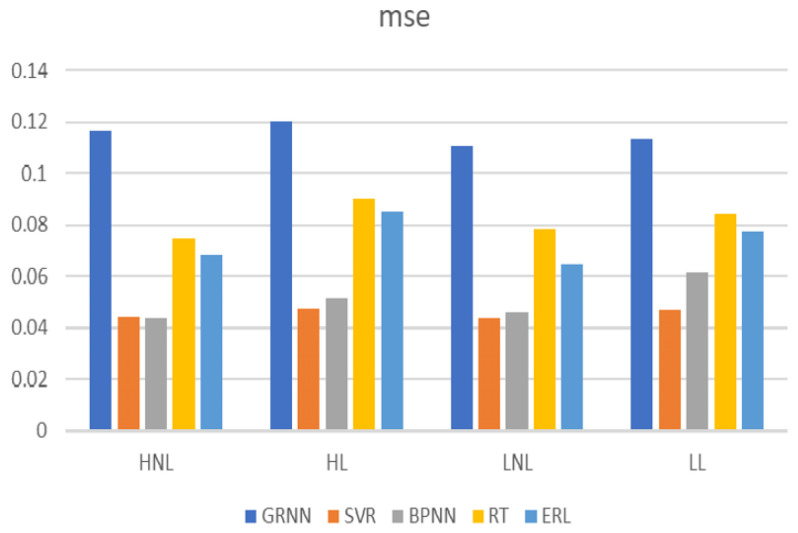
Mean square error of the different regression algorithms.

The chosen window width for the superimposed window in this paper was 130 signal points. The results are depicted in Figure 15, Figure 16, Figure 17, Figure 18, Figure 19 and Figure 20.

### 5.5. BPNN

**Figure 21:**
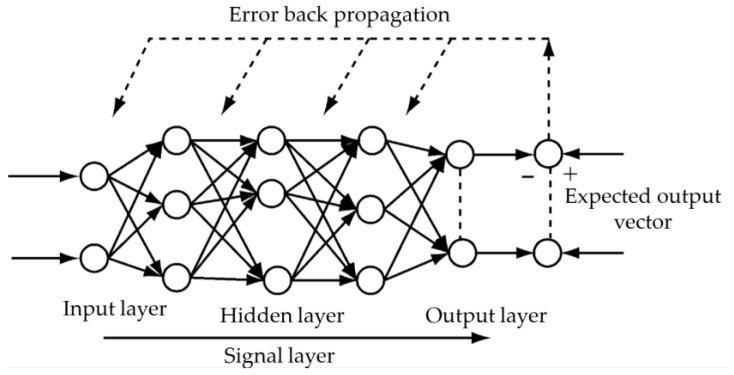
BPNN schematic.

An artificial neural network (ANN) is an algorithm designed and developed to simulate the structure and function of the human brain’s neural network. It consists of neurons connected by adjustable weights and exhibits a good adaptive learning ability. Among various neural network algorithms, the back propagation neural network (BPNN) is the most widely applied. Its structure is illustrated in Figure 21, which consists of an input layer, hidden layer, and output layer. The input signal is propagated in the forward direction through the layers with adjustable weights, while the error between the output and the desired pattern obtained by the output layer is transmitted back in the reverse direction through each layer using its quantized loss function. Simultaneously, the weights and thresholds of each layer are modified and updated to obtain the output closest to the label [37].

## 6. Control

According to the grading of muscle strength, limbs with a muscle strength below grade two are unable to resist gravity, especially in the initial stage after a stroke where the patient’s upper limb nerves and muscles may be damaged, resulting in a lack of active power. In this early rehabilitation stage, passive training is prioritized to improve muscle tone, reduce muscle spasms, and guide the affected limb towards active rehabilitation. The process of passive control involves operating the system as planned, processing the output information, and solving any deviations that may occur.

As the stroke progresses, the upper limb may regain some motor ability, and the upper limb rehabilitation robot transitions from leading the movements to following the patient’s movements. The focus then shifts to active rehabilitation, with an emphasis on human–machine interaction. Establishing a closed-loop pathway between the human and the machine can improve the effectiveness of rehabilitation in the later stages and restore normal physiological activities of the upper limb.

**Figure 22:**
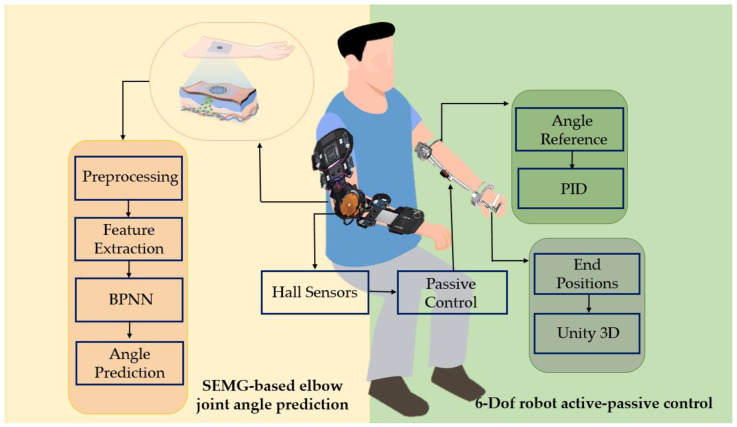
Schematic of the overall control strategy.

Based on the above rehabilitation perspective, this paper proposed three control modes, as depicted in the Figure 22.

1. Passive mode controlled by the PID (proportional-integral-derivative) to guide the initial rehabilitation process.
2. Active rehabilitation, where the affected side was driven by the healthy side, allowing for coordinated movement between both sides.
3. Interactive control, enabling the patient to move autonomously, fostering more independent and self-driven rehabilitation activities.

### 6.1. PID

The PID control is known for its simplicity in structure, stable operation, and widespread application. It has been extensively used in the fields of rehabilitation robotics and industrial control and remains one of the earliest and most popular control methods [38,39,40].

One of the advantages of PID control is that it does not heavily rely on the model of the control object. Satisfactory results can often be achieved by tuning the system parameters appropriately. In this study, a simple PID controller based on state feedback, as shown in Equation (10), was implemented to test the desired functionality of the developed exoskeleton robot. The trajectory of motion was controlled by manipulating the joint torques calculated by the PID controller. The PID controller effectively regulated the robot’s movements and ensured its desired performance during the rehabilitation exercises.

(10) $$\tau =K_{p}e+K_{V}\dot{e}+K_{I}\int edt$$

where

$$e=q_{d}-q,\dot{e}={\dot{q}}_{d}-\dot{q}$$

$q_{d}-q$ is the error between the desired joint position and the actual joint position.

${\dot{q}}_{d}-\dot{q}$ is the error between the desired joint vector and the actual joint vector.

*K****_P_***, *K****_I_***, and *K****_V_*** are the diagonal matrices for the proportional, integral gains, and derivative, respectively.

The reliability of such a controller depends on the proper selection of the proportional (*K****_P_***), derivative gains (*K****_V_***), and integral (*K****_I_***). In this paper, the PID gains were set for the controller. The gains chosen for the experiments were *K****_P_*** = diag (2200, 2000, 2200), *K****_I_*** = diag (50, 40, 50), and *K****_V_*** = diag (20, 18, 20).

**Figure 23:**
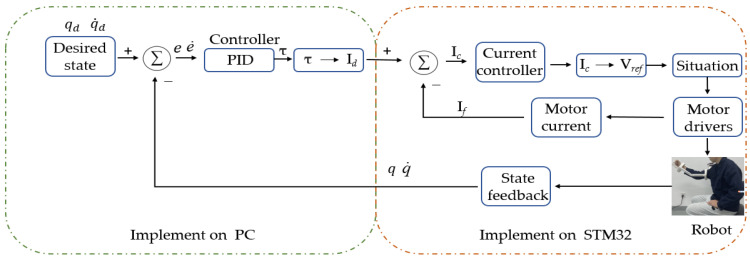
Principles of the PID control for upper limb rehabilitation robots.

As depicted in Figure 23, the exoskeleton upper limb rehabilitation robot employed joint-based control, wherein a predetermined position and velocity were input to the controller. Feedback from the position sensors was obtained to determine the actual position and velocity of the robot, allowing for the calculation of the error. The controller then estimated the necessary torque, angle, and angular velocity based on this information. Subsequently, using specific formulas, the obtained data were transformed into a motor current, which was referred to as the desired current. This control mechanism ensured that the robot’s movements were accurately controlled and aligned with the desired trajectory during the rehabilitation process.

### 6.2. Bilateral Control

Bilateral coordinated movement of the upper limb is an effective method for neural remodeling. Stroke patients often experience unilateral hemiparesis, and using the healthy limb to guide the rehabilitation training of the affected limb has shown positive results [41,42,43]. In a unilateral robotic system, the movement parameters of the healthy side are captured by sensors and used for mirror control of the affected limb. The electromyographic signals obtained from the healthy side provide valuable information about the motion intention of the upper limb. By utilizing a BP neural network, the features of the collected electromyographic signals can be extracted to establish a joint prediction model. This model effectively predicts the motion, enabling control of the mirror image of the affected limb.

**Figure 24:**
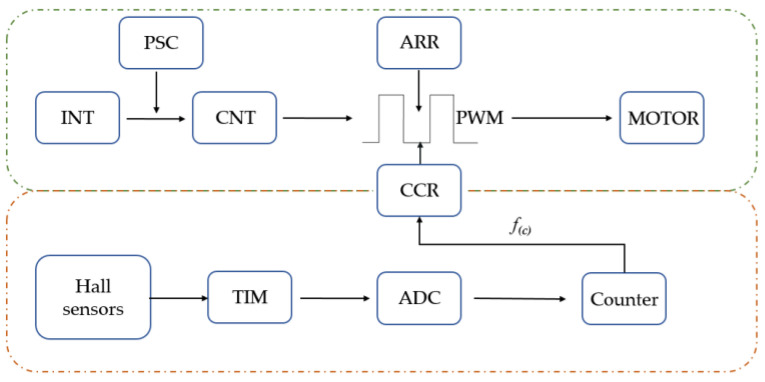
The healthy side drove the affected side control principle.

In this study, a Hall sensor was employed to extract the angle information from the healthy side’s elbow joint (the circuit principle is depicted in Figure 24). The BP neural network (BPNN) was then utilized to predict the angle of the elbow joint. The pre-scaler register (PSC) value was set to (7200^−1^), with an internal clock (INT) of 72 MHz, resulting in a counting time of 0.1 ms. The auto-load register (ARR) value was configured as (200^−1^), generating a counting interrupt period of 20 ms, which aligned with the required period for the servo drive. By using the output comparison function in the PWM mode, the duty cycle could be adjusted by modifying the value of the condition code register (CCR). The CCR value was derived from the Hall sensor through digital-to-analog conversion (ADC) using a specific formula transformation.

### 6.3. Interaction Control

Human–robot interaction plays a crucial role in the field of rehabilitation robotics, as it facilitates information exchange and interaction between humans and machines. This interaction becomes even more important as robots are required to work around people, enabling collaboration and seamless interaction. In essence, human–robot interaction aims to enhance machines’ ability to communicate effectively with humans. In the context of rehabilitation robot training, human–robot interaction leads to increased patient engagement and visualized training data.

Virtual reality technology is utilized to achieve human–robot interaction in rehabilitation robotics. This approach allows for more flexible and natural interactions between robots and humans, thereby enhancing the robot’s intelligence. As robot technology continues to advance, human–robot interaction is set to become a significant focus in future robot development [44].

**Figure 25:**
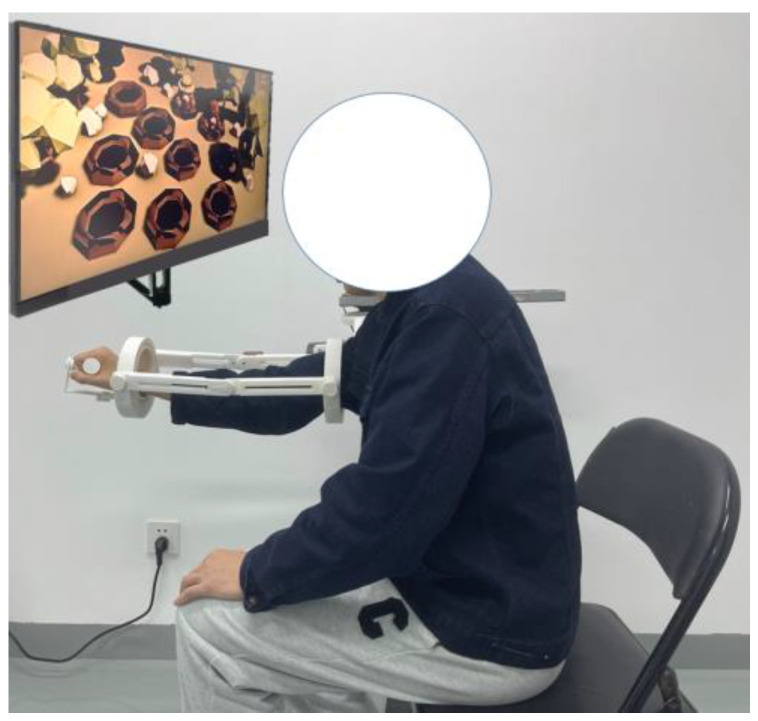
Unity3D-based game interactive interface.

In this paper, we developed a game scene using Unity3D and incorporated six-axis sensors to facilitate communication between the serial port and Unity3D. Through these sensors, game objects in the Unity3D scene could be controlled, enabling specific actions to be performed. Importantly, the patient retained full control during this process, and the robot did not provide assistance. The six-axis sensor was fixed at the end of the patient’s arm, and the arm position was mapped to the screen, allowing the training to be completed by extracting the end pose, as depicted in Figure 25.

## 7. Results

### 7.1. Results of the Elbow Joint Angle Measurement

**Figure 26:**
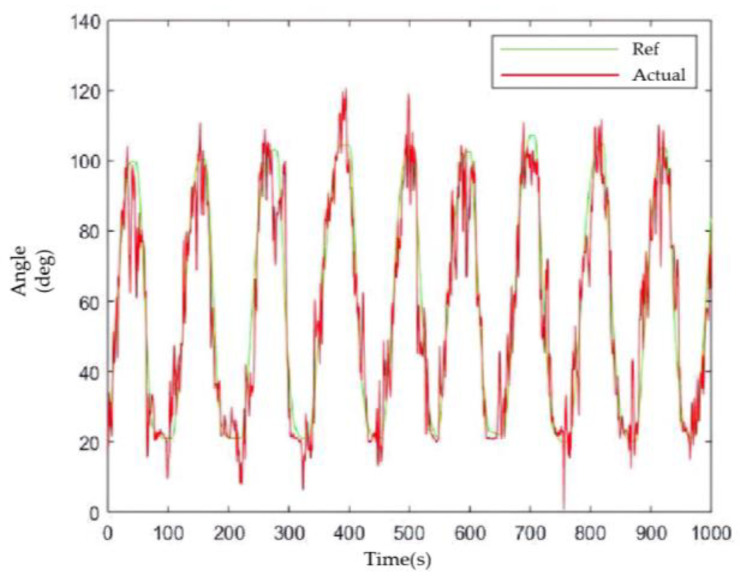
Actual vs. reference values for the elbow angle (where the red solid line represents the actual angle of the elbow joint predicted with the BPNN and the green solid line represents the reference angle).

Combined with the conclusions of the feature extraction in Chapter 5, the fifth order AR model coefficients were extracted from the collected sEMG. The BP neural network algorithm was then utilized for the quantitative modeling of the sEMG and elbow angle, with the number of neurons in the hidden layer set to 40. The waveforms of the actual (predicted) elbow angle and the reference (ideal) angle are depicted in Figure 26, where the red solid line represents the actual angle of the elbow joint, and the green solid line represents the reference angle.

The linear regression relationship between the refence value and the actual value showed a high R value of 94.41%. Additionally, the average error between the two angles was approximately 4° with a MSE of 7.2°^2^.When compared with the other literature that reported angle prediction errors in the range of 5~8° [45], the model established in this paper demonstrated a better prediction effect.

### 7.2. Control Results

**Figure 27:**
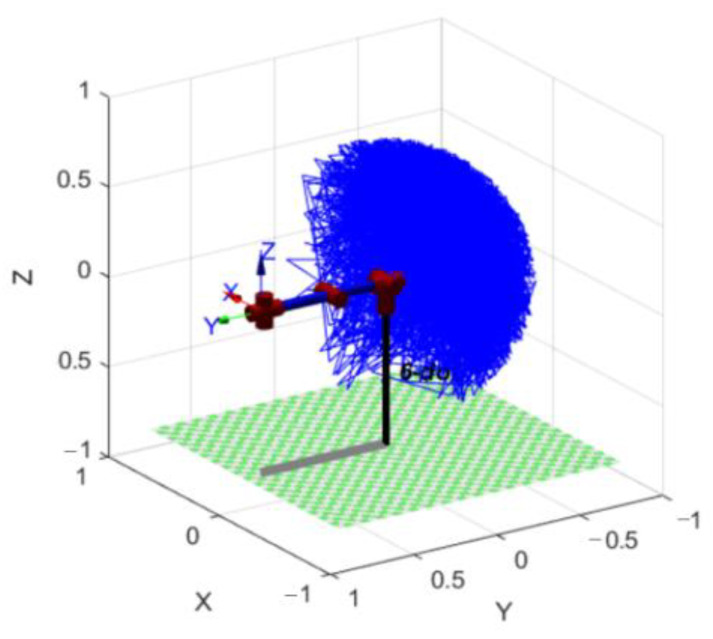
Robot spatial motion trajectory.

This experiment aimed to analyze the motion of a single joint, and in order to validate the experimental results, it was analyzed with respect to PID control and healthy side-driven affected side control. The robot kinematic model was established using the Link and Serial Link functions in the MATLAB Robotics Toolbox. The link parameters of the upper limb rehabilitation robot were input to obtain its kinematic space, as illustrated in Figure 27, and the results indicated that the joints could achieve sufficient movement.

**Figure 28:**
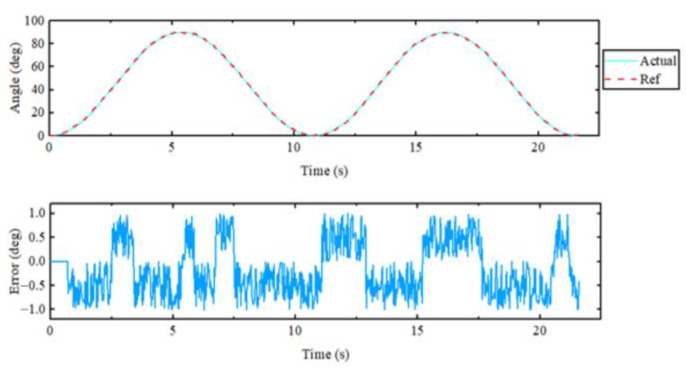
Positional error plot of the single-joint flexion and extension motion of the shoulder joint.

**Figure 29:**
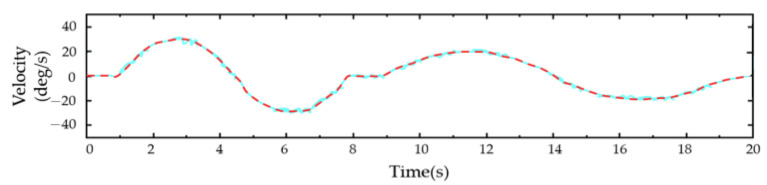
Velocity–time diagram of the shoulder unicompartmental flexion and extension.

**Figure 30:**
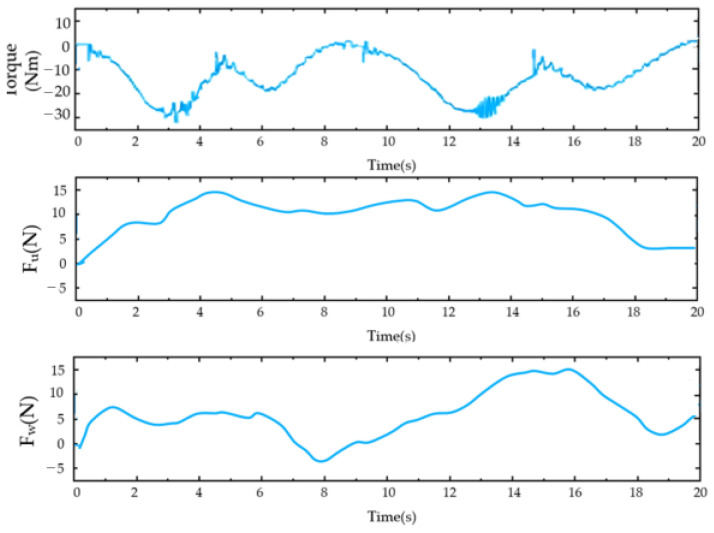
Motor output torque and limb force during the shoulder joint single-joint flexion–extension exercise (from top to bottom: the motor output torque, force measured by the shoulder cuff module, and wrist force).

**Figure 31:**
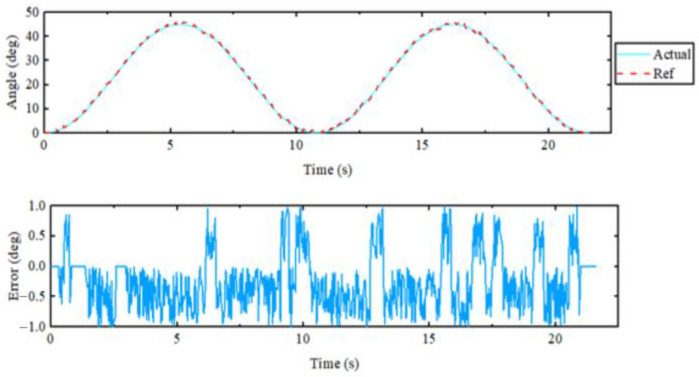
Shoulder internal and external rotation single-joint training position error chart.

**Figure 32:**
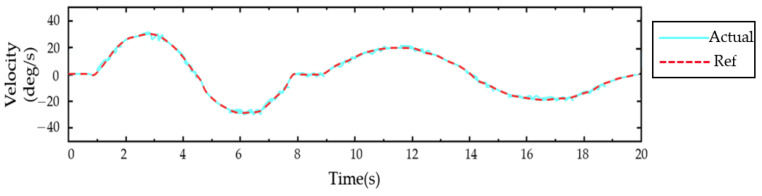
Single-joint velocity–time diagrams for shoulder internal and external rotation.

**Figure 33:**
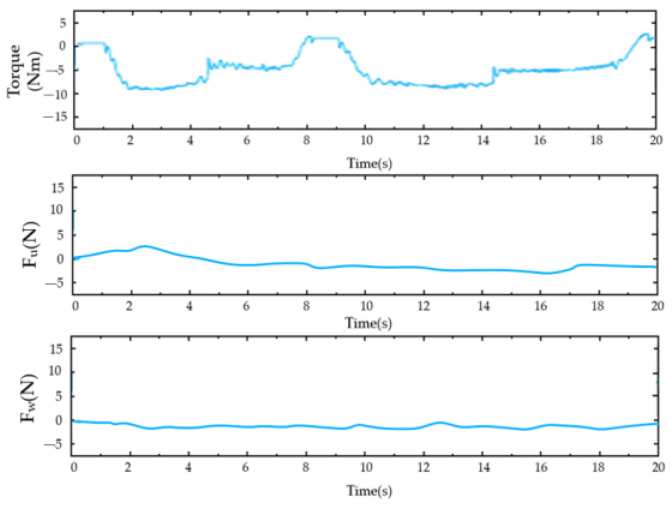
Motion output torque and limb strength during the unicompartmental rotational internal and external movements of the shoulder joint (from top to bottom: the motion output torque, strength measured by the rotator cuff module, and wrist strength).

**Figure 34:**
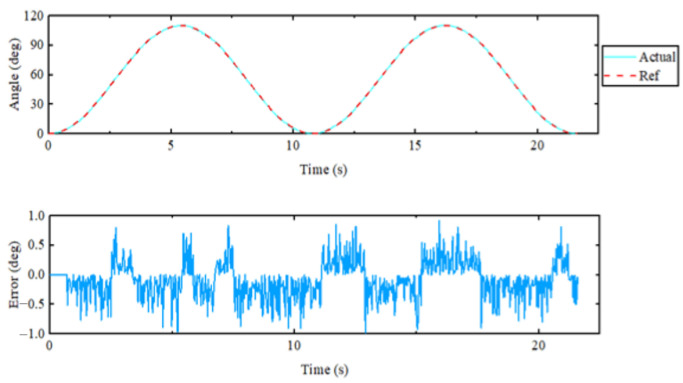
Elbow flexion extension single-joint exercise.

**Figure 35:**
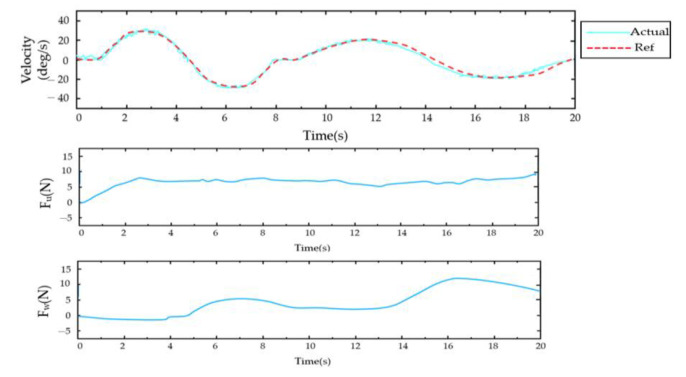
Velocity profiles and limb strength during the elbow uni-joint flexion–extension movements (from top to bottom: the velocity–time relationships, strength measured by the rotator cuff module, and wrist strength).

**Figure 36:**
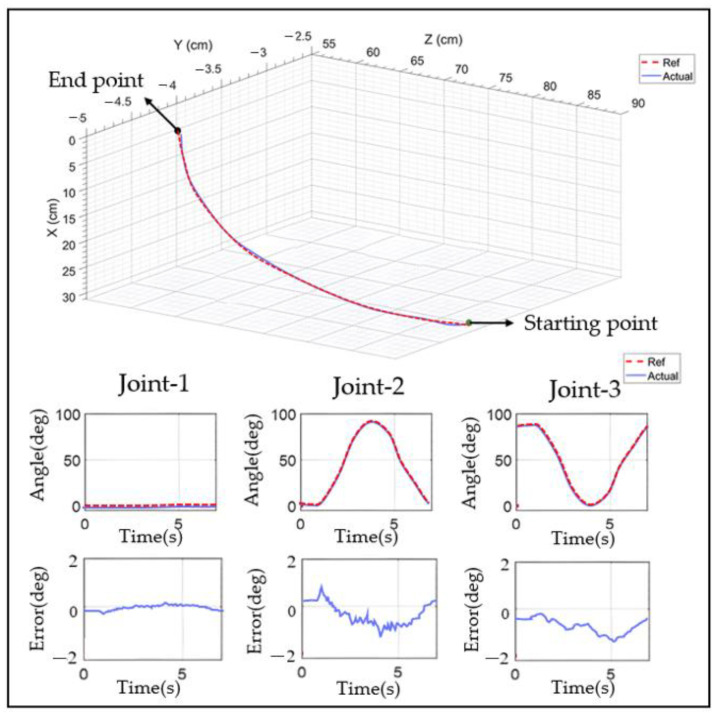
Sagittal plane reach trajectory diagram.

**Figure 37:**
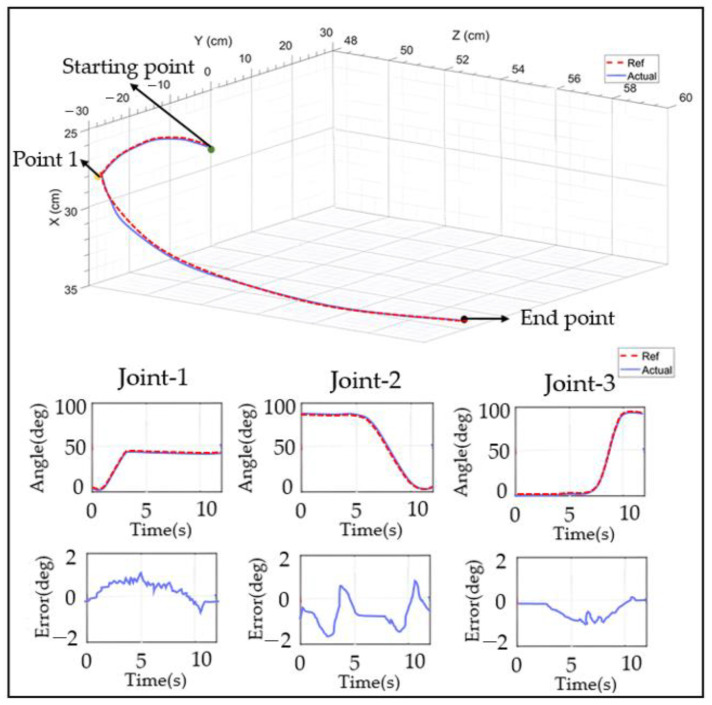
Receiving object trajectory movement diagram.

For the PID control (Figure 28, Figure 29, Figure 30, Figure 31, Figure 32, Figure 33, Figure 34, Figure 35, Figure 36 and Figure 37), the plots of the joint position versus time and the error plots of the reference position versus the actual position are provided. The red dashed lines represent the reference values for the position and velocity tracking, while the blue solid lines indicate the actual values.

For the shoulder flexion–extension movement, the joint was initially positioned at zero degrees and then extended to 85° before returning to 0°. The same movement was repeated once more. From the top curve shown in Figure 28 and Figure 29, it was evident that the actual position closely aligned with the reference position, indicating that the proposed exoskeleton robot accurately followed the given reference position. The maximum error in position tracking was only 1.02°, demonstrating that the controller exhibited an excellent tracking performance. Additionally, the maximum velocity during the repetitive motion was approximately 25.3 degrees per second.

Figure 29 depicts the velocity–time curves during the unicompartmental movements of the shoulder joint. In both flexion–extension motions, the velocity change in the first round-trip motion was greater than in the second one. Throughout the movement, there were fluctuations, but these were characterized by minimal errors.

As shown in Figure 30, the top image illustrates the output torque of the reducer during this movement. The middle image displays the measured force of the rotating cuff at the shoulder joint, while the bottom image shows the measured force of the rotating cuff at the wrist joint. Notably, the gearbox operated within the rated torque range throughout the process. The force sensor recorded the highest interaction between the subject and the system during the vertical flexion of the shoulder joint.

For the inward and outward shoulder rotation, the experiment began with all the joints in the zero position. The shoulder was then horizontally rotated outward to 45° and returned to 0°. This motion was repeated at the same speed. The experimental results are presented in Figure 31, Figure 32 and Figure 33. The maximum error in position tracking was approximately 1.21°, indicating a good tracking performance of the controller. The maximum speed during the repetitive motion was 13 degrees per second. However, it was noteworthy that from the error plot, it could be observed that the position sensor data fluctuated significantly from the ideal data error when the subject performed rotational motion on the horizontal plane. Since only one force transducer was situated in the rotating cuff assembly to measure the forces in the anterior sagittal plane, the interaction between the force transducer and the subject was minimized when the shoulder joint was rotated inward and outward.

For the elbow flexion–extension motion, the elbow joint was repeatedly extended to 95° and then returned to 0°. The experimental results are displayed in Figure 34 and Figure 35. From the top curve in the figure, it was evident that the actual position and the reference position almost coincided with each other, and the maximum error in position tracking was 0.93°, indicating an excellent tracking performance of the controller. The maximum speed during the repetitive motion was 26 degrees per second. Since the rudder was chosen as the drive for the elbow joint, the rated torque was continuously output throughout the operation. The force transducer of the rotating cuff interacted most with the subject when the elbow joint was vertically flexed.

For simultaneous shoulder and elbow joint movements, the first movement replicated a forward-reaching motion, starting from the initial position (all the joints at 0° and the elbow at 90°), moving to the reaching position (the shoulder extended to 90° and the elbow extended to 90°), and then returning to the initial position. As shown in Figure 36, the results indicated that the developed exoskeleton robot followed the reference trajectory. From the figure, it can be seen that the positional errors of all the joints remained below 2°, with the elbow joint having the largest error (1.65°).

The diagonal catch movement consisted of flexion–extension and the inward and outward rotation of the shoulder joint, as well as flexion–extension of the elbow joint. The movement began with the elbow and shoulder joints internally and externally rotated at 0°. First, the shoulder joint was internally rotated to 45° and then was maintained at this angle until the end of the movement (located at Point 1). Subsequently, the shoulder joint was internally retracted from 90° to 0°, and the elbow joint was rotated from 0° to 90° before the movement concluded. The experimental results are shown in Figure 37. It was observed that the maximum error between the reference and actual positions of joint two was approximately 1.83°.

Due to the unilateral disability of hemiplegic patients, special consideration should be given to the mutual coordination of bilateral limbs in bilateral rehabilitation training. In this study, we chose to use the healthy upper limb to drive the affected side as a rehabilitation task to promote bilateral coordination. The goal of this task was to achieve better coordination by equalizing the angle of motion of the elbow joints of both upper limbs. However, for hemiplegic patients, accomplishing the equivalent output force of the affected elbow joint without any assistance or feedback was challenging.

**Figure 38:**
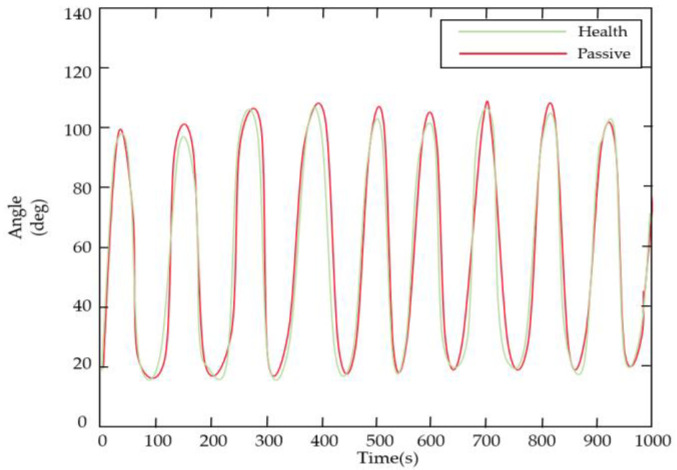

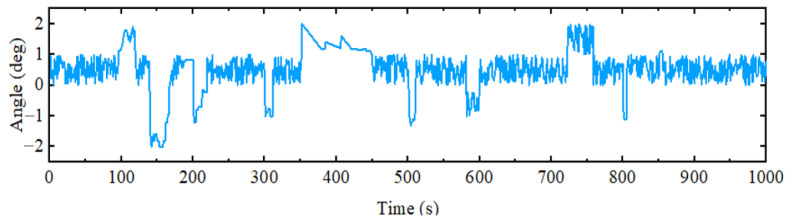
The healthy side of the elbow drove the affected side.

It was previously mentioned that there were still errors in angle prediction using BPNN, and there was some delay in the system. Therefore, sEMG was not introduced as a parameter for the healthy side to drive the affected side’s movement. It was more reliable to have the patient wear the elbow rehabilitator on the healthy side and use the Hall sensor to obtain the joint angle. To demonstrate the stability of this approach, the subjects were asked to complete a single elbow joint exercise repeatedly for 1000 s. The results of the experiment are shown in Figure 38, with a mean error of 1.56 degrees. This indicated a relatively small deviation between the desired and actual elbow joint angles during the repetitive motion, suggesting that the task was stable and achievable for the participants.

## 8. Conclusions

Through research on rehabilitation robots in recent years, significant breakthroughs have been made by research institutes and universities in the field of upper limb rehabilitation robots. The development trend has shifted from low degrees of freedom to high degrees of freedom, and from traditional control to artificial intelligence control. In designing upper limb rehabilitation robots, it is essential to consider the precise torque required for limb operation, taking into account the differences in the structure, control, and specific treatments.

This study presented the development of a six-degree-of-freedom (6-DOF) upper limb rehabilitation robot with three control modes, an end-pose extraction module, and a mechanical joint capable of rotation around the center. A hybrid approach was utilized to derive the kinematic equations of the developed robot. The implementation of PID control algorithms allowed for passive control of the patient during the prehabilitation period.

The experimental results demonstrated that the robot exhibited low tracking error in joint motion. For patient active rehabilitation in the middle stage, bilateral control was employed, enabling patients to actively participate and improve their training efficiency. Before this, an eight-channel elbow angle prediction system was developed, and the experiments confirmed the effectiveness of using BPNN for prediction. The patients wore an elbow rehabilitator on the healthy side and the upper extremity rehabilitation robot on the affected side, utilizing the healthy side to drive the affected side. During repetitive tracking training, the affected side’s upper limb was able to achieve basic following, although a slight delay was observed in the data transmission from the Hall sensor to the register.

For the interaction control, a Unity3D-based game system was developed, enabling the patients to participate in the game based on the position of their end arm. This approach increased the patients’ interest in rehabilitation and allowed for a better visualization of the rehabilitation training data. Future work may involve the experimental validation of active rehabilitation and the incorporation of impedance in the interaction control.

To address the issue of delayed data transmission, several strategies can be employed. Firstly, elevating the sampling rate can enhance the data density, yielding finer and more detailed information, consequently bolstering the temporal resolution of events. Secondly, data interpolation and filtering can be employed. While increasing the sampling rate may augment the data volume, filtering out high-frequency noise and erratic data can alleviate delays. Thirdly, advanced control algorithms, such as ahead-lag control or model predictive control, can be implemented. These algorithms, by predicting future events, enable proactive system responses, reducing reliance on real-time data and mitigating data transmission delays.

In the future, our research will emphasize the comprehensive evaluation of the rehabilitation robot efficacy on hemiplegic patients, both in the short-term and long-term rehabilitation phases. We aim to investigate the effectiveness of upper limb rehabilitation robots combined with multimodal control for patients across various life stages. Our focus will also extend to enhancing the interaction design for Unity3D, with the intention of creating games grounded in the patients’ daily activities, allowing us to assess their impact on the rehabilitation process and overall effectiveness.

## Figures and Tables

**Table 1:** Upper limb joint muscles and mobility.

Joint	Joint No.	DOF	Main Muscles	Rom
Shoulder	J2	Flexion	Coracobrachialis	70~90°
		Extension	Latissimus dorsi	40~90°
	J1	Internal rotation	Latissimus dorsi	70~90°
		External rotation	Infraspinatus	40~50°
Elbow	J4	Flexion	Biceps	135~150°
		Extension	Triceps	0~10°
Forearm	J5/J3/J2′	External rotation	Pronator teres	80~90°
		Internal rotation	Supinator muscle	80~90°
Wrist	J6	Flexion	Flexor carpi ulnaris	50~60°
		Extension	Extensor carpi ulnaris	30~60°
	J6′	Adduction	Flexor carpi radialis	30~40°
		Abduction	Flexor/Extensor carpi ulnaris	25~30°

**Table 2:** Design specifications and selected components of the rehabilitation robot.

Degrees of Freedom							
Active				Passive			
3				3			
Ranges of motion/joints’ limits (degrees)							
Joint-J1	Joint-J2		Joint-J4	Joint-J5/3/2′	Joint-J6		Joint-J6′
−40 to 90	−45 to 90		0 to 135	−90 to 80	−60 to 40		−40 to 30
**Fabrication**							
Material	Aluminum 6061, stainless steel 304, plastic (photosensitive resins and polylactic acid)						
Fabrication process	Modeling, material selection, CNC machining, lathe turning, 3D printing, module assembly, circuit assembly						
**Actuators**							
Location		Joint-J1		Joint-J2		Joint-J4	
Motors		Speed reducer DH-03X		Speed reducer DH-03X		Steering engine DS5160, 12.6 W	
Operating voltage		12–24 V		12–24 V		8.4 V	
Nominal speed		0.12–0.24 s/60°		0.12–0.24 s/60°		0.08 s/60°	
Nominal torque		11–38 N·m		11–38 N·m		7 N·m	
Weight		595 g		595 g		158 g	
Motor drivers		KSMA-03X DHMCU				-	
Motor driver current rating		5–12 A				-	
Motor driver input		12–24 V DC power supply				-	
Motor driver feedback		Current sense, Hall sensor pulses				-	
**Damping hinges**							
Location				Joint-J6/J6′			
Nominal torque				0.7 N·m			
Material				Stainless steel 304			
Weight				25 g			
Thicknesses				2 mm			
Angle of activity				0–250°			
**Upper arm rotating cuff assembly**							
Location			J3/J2′		J5		
Inside diameter			65 mm		120 mm		
External diameter			80 mm		140 mm		
Pressure sensors				RDF-6			
**Embedded hardware circuitry**							
Circuit board				STM32F407ZGT6			
Hall sensors				P3302(0.3%F.S.)			
Position sensor				MPU6050			
Myoelectric sensors				DFRobot in cooperation with OYMotion			
Charger interface				DC5.5 × 2.5			
Network interface				RJ45			
Electrode patch				X-1, Xunda Radio Co.(Hangzhou, China)			

**Table 3:** Mass inertia properties of the proposed exoskeleton system.

Module	Length (mm)	Weight (kg)	Center of Gravity (mm)			Moment of Inertia I at CG (kg·mm^2^) (10^3^)		
			CG_X_	CG_Y_	CG_Z_	I_XX_	I_YY_	I_ZZ_
Shoulder joint	247	3.2	−4.3	−187.4	−46.1	102.1	25.3	79.8
Elbow joint	330	1.8	3.7	−9.5	8.9	70.6	24.7	21.3
Wrist joint	15 ± 5	0.7	22.8	0	−70.9	0.2	0.8	0.8

**Table 4:** DH parameter table.

*i*	*θ_i_*	*d_i_*	*a_i_*	*α_i_*
1	*θ*_1_ (−90°)	*d*_1_	0	90°
2	*θ*_2_ (−90°)	0	0	90°
3	*θ*_3_ (180°)	*d*_3_	0	90°
4	*θ*_4_ (−90°)	0	*a*_4_	−90°
5	*θ*_5_ (90°)	0	0	90°
6	*θ*_6_ (0°)	*d*_6_	0	90°

**Table 5:** Noise source and frequency band.

Number	Noise Sources	Frequency (Hz)	Affect
1	Industrial frequency interference	50	One of the main noises
2	Cardiac interference	0–30	One of the main noises
3	Non-muscle collection	0–15	Very low frequency interference

**Table 6:** Heights and weights of the experimenters.

Members	Height (cm)	Weight (Kg)	Age
M1	174	75	25
M2	176	82	35
M3	180	71	26
M4	183	75	25
W1	166	57	22
W2	170	54	24

## Data Availability

All the test data mentioned in this paper will be made available upon request from the corresponding author with appropriate justification.
